# West Nile Virus State of the Art Report of MALWEST Project

**DOI:** 10.3390/ijerph10126534

**Published:** 2013-12-02

**Authors:** Andriani Marka, Alexandros Diamantidis, Anna Papa, George Valiakos, Serafeim C. Chaintoutis, Dimitrios Doukas, Persefoni Tserkezou, Alexios Giannakopoulos, Konstantinos Papaspyropoulos, Eleni Patsoula, Evangelos Badieritakis, Agoritsa Baka, Maria Tseroni, Danai Pervanidou, Nikos T. Papadopoulos, George Koliopoulos, Dimitrios Tontis, Chrysostomos I. Dovas, Charalambos Billinis, Athanassios Tsakris, Jenny Kremastinou, Christos Hadjichristodoulou

**Affiliations:** 1 Department of Hygiene and Epidemiology, Faculty of Medicine, University of Thessaly, Larissa 41222, Greece; E-mails: anmar@med.uth.gr (A.M.); petserke@med.uth.gr (P.T.); 2 Laboratory of Entomology and Agricultural Zoology, School of Agricultural Sciences, University of Thessaly, Volos 38446, Greece; E-mails: alexdiamandidis@yahoo.gr (A.D.); nikopap@agr.uth.gr (N.T.P.); 3 National Reference Center for Arboviruses, Department of Microbiology, School of Medicine, Aristotle University of Thessaloniki, Thessaloniki 54124, Greece; E-mail: annap@med.auth.gr; 4 Laboratory of Microbiology and Parasitology, Faculty of Veterinary Medicine, University of Thessaly, Karditsa 43100, Greece; E-mails: georgevaliakos@gmail.com (G.V); alexiosg@yahoo.gr (A.G.); kodafype@for.auth.gr (K.P.); billinis@vet.uth.gr (C.B.); 5 Laboratory of Microbiology and Infectious Diseases, School of Veterinary Medicine, Faculty of Health Sciences, Aristotle University of Thessaloniki, Thessaloniki 54124, Greece; E-mails: schainto@vet.auth.gr (S.C.C.); dovas@vet.auth.gr (C.I.D.); 6 Laboratory of Pathology, Faculty of Veterinary Medicine, University of Thessaly, Karditsa 43100, Greece; E-mails: ddoukas@vet.uth.gr (D.D.); dtontis@vet.uth.gr (D.T.); 7 Department of Parasitology, Entomology and Tropical Diseases, National School of Public Health, Athens 11521, Greece; E-mail: epatsoula@esdy.edu.gr; 8 Laboratory of Biological Control of Pesticides, Benaki Phytopathological Institute, Athens 14561, Greece; E-mails: ebadieritakis@yahoo.gr (E.B.); g.koliopoulos@bpi.gr (G.K.); 9 Hellenic Centre for Disease Control and Prevention (KEELPNO), Athens 15123, Greece; E-mails: baka@keelpno.gr (A.B.); mariatseroni@googlemail.com (M.T.); pervanidou@keelpno.gr (D.P.); president@keelpno.gr (J.K.); 10 Department of Microbiology, Faculty of Medicine, University of Athens, Athens 11527, Greece; E-mail: atsakris@med.uoa.gr

**Keywords:** West Nile virus, Greece, surveillance, control, epidemiology, diagnosis, mosquito, bird, risk assessment, GIS

## Abstract

During the last three years Greece is experiencing the emergence of West Nile virus (WNV) epidemics. Within this framework, an integrated surveillance and control programme (MALWEST project) with thirteen associate partners was launched aiming to investigate the disease and suggest appropriate interventions. One out of seven work packages of the project is dedicated to the State of the Art report for WNV. Three expert working groups on humans, animals and mosquitoes were established. Medical databases (PubMed, Scopus) were searched together with websites: e.g., WHO, CDC, ECDC. In total, 1,092 relevant articles were initially identified and 258 of them were finally included as references regarding the current knowledge about WNV, along with 36 additional sources (conference papers, reports, book chapters). The review is divided in three sections according to the fields of interest: (1) WNV in humans (epidemiology, molecular characteristics, transmission, diagnosis, treatment, prevention, surveillance); (2) WNV in animals (epidemiological and transmission characteristics concerning birds, horses, reptiles and other animal species) and (3) WNV in mosquitoes (control, surveillance). Finally, some examples of integrated surveillance programmes are presented. The introduction and establishment of the disease in Greece and other European countries further emphasizes the need for thorough research and broadening of our knowledge on this viral pathogen.

## 1. Introduction

West Nile virus (WNV) is a mosquito-borne flavivirus causing to humans a variety of symptoms: from an asymptomatic infection to severe and even fatal encephalitis [1]. Wild and domestic birds are reservoirs of the virus; transmission takes place via the *Culex* mosquito-vectors towards dead-end hosts (human, equine, other mammals or vertebrates). Although most patients with WNV infections do not present any symptoms or display mild flu-like symptoms, there are few people that develop severe illness involving the central nervous system (CNS) including meningitis, encephalitis, acute flaccid paralysis [2]. People over 50 years old, immunosuppressed or with chronic diseases are at higher risk to present a severe disease, sometimes with fatal outcome [3]. In 1999, WNV emerged in the United States of America (USA) [4] and since has become distributed in the whole country affecting humans, birds and equines, while in Europe, the first large-scale epidemic took place in 1996 in Romania [5,6].

In Greece, human WNV infections were laboratory confirmed for the first time in 2010, in the form of an outbreak, mainly in the northern part of the country, with 262 recorded cases and 35 deaths, while in 2011, 100 cases and nine deaths were reported [7]. In October 2011, the Hellenic Ministry of Health approved the two-year funding of the *“Integrated surveillance and control programme for West Nile virus and malaria in Greece”* (MALWEST) through the Operational Programme “Human Resources Development” of National Strategic Reference Framework (NSRF) 2007–2013. Thirteen institutions and universities are the associate partners of this project (including the Hellenic Center for Disease Control and Prevention and four Greek Universities). 

The main objectives of the programme, regarding WNV, are: (1) the mapping of the WNV activity and its impact on public health; (2) the detection of the geographic regions with the greatest risk and the development of risk assessment tools by using Geographical Information Systems (GIS);(3) the prediction of spreading of the disease and 4) the assessment of appropriate interventions. One out of the seven work packages of the project is entitled “State of the Art for WNV surveillance and control”, which is dedicated to a systematic review of the current knowledge available on WNV in order to be used as a basis for the project activities.

The establishment of the disease in Greece in the form of epidemics during the last three years, the knowledge of transmission dynamics of the virus and the complex epidemiological chain, in which the virus is involved, were the main factors prompting to deepen our knowledge in the field of WNV and prepare the present State of the Art report. 

## 2. Methods

Three expert working groups were established within the MALWEST project selecting participants according to their scientific field: (1) WNV in humans, epidemiology, surveillance and risk assessment; (2) WNV in animals and (3) WNV in mosquitoes. Through continuous editorial meetings, the methodology, context and structure of the review were decided. 

The bibliographic search was made using internet search engines and medical databases as Medline (PubMed) and Scopus, as well as through the websites of the World Health Organization (WHO), the Center for Disease Control and Prevention (CDC), the European Center for Disease Control and Prevention (ECDC) and other relevant web pages. The keywords used were: “West Nile virus” combined with terms like “West Nile fever”, “case”, “transmission”, “epidemics”, “epidemiology”, “molecular epidemiology”, “diagnosis”, “PCR”, “ELISA”, “detection”, “seroprevalence”, “IgM”, “IgG”, “treatment”, “prevention”, “vaccine”, “control”, “surveillance”, “risk assessment”, “mosquito”, “mosquito management”, “pesticides”, “larvicides”, “equine”, “horse”, “bird”, “chicken”, “pigeon”, “sentinel”, “seroconversion”, “Culex”, “Culicidae”, “breeding sites”, “lakes”, “wetlands”, “channels”, “GIS”. During the first editorial meeting, 454, 315 and 323 journal articles abstracts were found for the sections of humans, animals and mosquitoes, respectively. Under the experts’ supervision in each working group, the most important and representative articles were selected, and finally the 258 full texts used for the State of the Art report were: 18 for all the parts of the review and 152, 48 and 40 for WNV in humans, animals and mosquitoes, respectively. In addition, 10 books, eight book chapters, eight web sites, six reports, three conference papers and one PhD thesis were included.

## 3. WNV in Humans

### 3.1. General Aspects—Molecular Epidemiology

#### 3.1.1. Taxonomy

WNV is classified as a member of the genus Flavivirus in the *Flaviviridae* family, which contains more than 70 viruses, with approximately 40 of them being human pathogens (e.g., dengue fever viruses, tick-borne encephalitis virus, Japanese encephalitis virus, Yellow fever virus, *etc.*) [8]. On the basis of serological cross-reactivity, Flavivirus genus is subdivided into 12 antigenic serocompexes, and WNV belongs to the Japanese encephalitis serocomplex. 

WNV strains cluster into at least five different lineages [9]. Lineage 1 is distributed almost worldwide, while lineage 2 was initially thought to be restricted to sub-Saharan Africa and Madagascar. However, since 2004, lineage 2 strains caused large outbreaks in humans in several European countries [10]. Lineage 3 consists of Rabensburg virus, isolated from *Culex pipiens* in 1997 in the Czech Republic, near the Austrian border [11]. Lineage 4 comprises isolates from Caucasus region in Russia, first detected in 1988 from a *Dermacentor* tick, and later isolated from mosquitoes. Lineage 5 (or clade 1c) contains isolates from India obtained from humans and mosquitoes. A putative lineage 6 consists of a sequence detected in 2006 in *Culex pipiens* mosquitoes in Southern Spain [12], while Koutango virus isolated in Senegal might represent a seventh lineage, although it is currently categorized as a separate species [13]. 

#### 3.1.2. The Virion

WNV virions are small spherical enveloped particles, with an icosahedral structure of approximately 50 nm in diameter. They contain a single-stranded, positive-sense RNA genome of approximately 11 kb, with 51% guanine-cytosine content. Almost 93% of the WNV genome is encoded, forming a single polyprotein of 3,400 amino acids. Following cleavage by viral and host proteases, 10 mature proteins are generated: three structural (core protein-C, pre-membrane prM/membrane protein-M and envelope protein E) and seven non-structural (NS) proteins (NS1, NS2a, NS2b, NS3, NS4a, NS4b and NS5). The C protein binds the viral RNA, the prM protein blocks premature viral fusion and the E protein mediates viral attachment, membrane fusion and viral assembly [14]. E protein contains 12 cysteines involved in the formation of intramolecular disulfide bonds and the production of homodimers. It consists of three domains: domain III is an immunoglobulin which is thought to be involved in the interactions between virions and host cells to enable the virus entrance, domain II contains a conserved region of 13 hydrophobic amino acids that form an internal fusion loop necessary for fusion, while domain I links domains II and III [15]. E protein promotes the production of neutralizing antibodies [16]. The NS proteins are involved at various stages of the transcription, translation, replication and assembly of the virus: NS1 and NS4a are involved in the replication of the virus, NS2a is involved in assembly and release of the virion, NS2b and NS3 have proteolytic properties, NS4b enhances the helicase activity, while NS5 encodes the RNA-dependent RNA polymerase and a methyltransferase [17]. Furthermore, the NS proteins modulate the interferon signaling cascade in vertebrate hosts [18]. The E protein is the major antigenic protein, but it seems that prM, NS1, NS3 and NS5 have also antigenic properties, while NS1 is often used as antigen for diagnostic enzyme-linked immunosorbent assay (ELISA) methods [19].

After virus attachment and entry into the target cells of the host via receptor-mediated endocytosis, the viral RNA is released into the cells, and it is translated by the host machinery; the viral genome is replicated, and new viral RNA and proteins are synthesized, which are further processed and packaged to form new virions, ready to be released from the cell by exocytosis and/or budding at the plasma membrane [17]. 

#### 3.1.3. Molecular Epidemiology

As mentioned above, WNV strains cluster into at least five genetic lineages; however, most of the reported pathogenic strains belong into the major lineages 1 and 2. It is currently known that WNV lineage 1 includes strains from Africa, Asia, Europe, America (clade 1a) and Australia (clade 1b). Especially in America, WNV was first reported in the US in 1999 (genotype NY99) [20,21]; in 2001 a new strain (WN02) containing the A159V substitution in E protein rapidly replaced the NY99 genotype [22], probably due to its ability to disseminate more efficiently in domestic *Culex pipiens* and *Culex tarsalis* mosquitoes as compared to the NY99 genotype [23,24], while a third genotype had emerged in the Southwestern USA after 2003, termed genotype SW/WN03 [25]. Phylogenetic studies based on full genome sequences showed that WNV clade 1a originated in sub-Saharan Africa in the early 20th century, and then spread northwards to both Eastern and Western European countries since the late 1970s, following the routes of the migratory birds, with white storks being probably implicated in the spread of WNV [26,27]. It is obvious that selective pressures impact viral population genetics, and genotypic and phenotypic changes which have occurred in the WNV genome as it adapts to novel environments [28].

**Figure 1:**
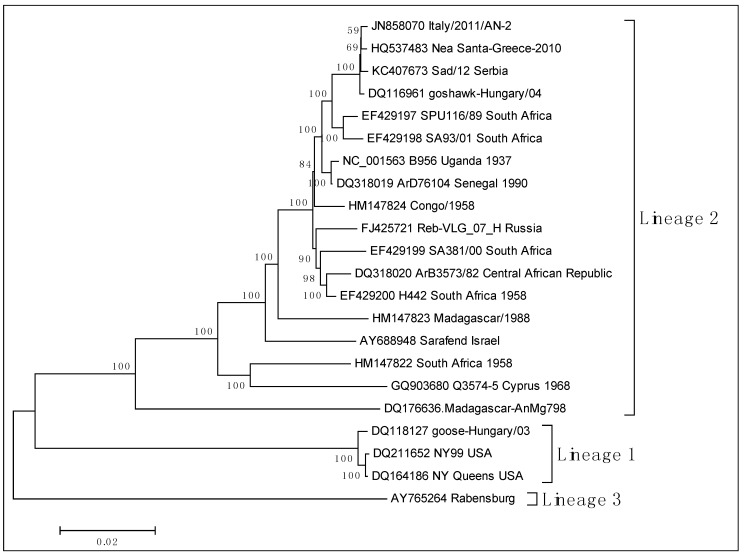
Neighbor-Joining phylogenetic tree (1,000 replicate trees, 10,278 positions in the final dataset) showing the clustering of the WNV strain Nea Santa-Greece-2010 into the WNV lineage 2. Evolutionary analyses were conducted in MEGA5 [41].

Since all outbreaks in Europe, Israel and America were caused by WNV lineage 1 strains, it was suggested that this is the pathogenic lineage, while WNV lineage 2 was considered as not pathogenic. However, recent studies in Africa showed that there are pathogenic strains belonging to this lineage [29,30,31]. In general, it seems that the role of WNV lineage 2 has been previously underestimated and that the virulence is associated with the specific strain rather than the lineage, the location or the year of isolation [29,30,31,32,33]. It is of interest that the prototype WNV strain B 956 initially isolated in 1937 in Uganda from the blood of a febrile female patient is classified in lineage 2. The first detection of WNV lineage 2 in Europe occurred in 2004 in a goshawk in Hungary [34]. Similar nucleotide sequences were obtained during the following years from birds of prey in Hungary, and in 2008, also in Austria. The first detection in humans occurred in 2007 in Russia [35]. Mild human cases have been reported later in Hungary and Austria [36,37,38]. The first evidence that the strain that caused the large outbreak in 2010 in Greece was a WNV lineage 2 strain, came when sequences obtained from *Culex pipiens* mosquitoes collected in Nea Santa village in Northern Greece (where a WNV human case was diagnosed) were found to be similar to lineage 2 strains from Africa and Hungary [39]. The genetic characterization of the complete genome of that strain (Nea Santa-Greece-2010, GenBank accession number HQ537483) showed the closest genetic relationship (99.6% nucleotide sequence identity) to the goshawk-Hungary-2004 strain (DQ116961) [40] (Figure 1).

It has been suggested that the increased virulence may be associated with the amino acid substitution H_249_P in the NS3 protein; the Greek strain was the only one of lineage 2 strains with proline in this position [40]. This specific substitution has been previously associated with increased pathogenicity of WNV lineage 1 strains in the American crows [42]. Almost identical sequences (containing the H_249_P) were obtained from clinical human cases [43,44] and from a Greek blood donor [45], as well as from sedentary wild birds [46] and sentinel chickens [47]. A similar WNV lineage 2 strain was detected in 2011 in Ancona, Italy [48] and it is currently known that both WNV lineages circulate in Italy [49]. Currently, WNV appears to be expanding its geographical range in Europe and causing increasing numbers of epidemics/outbreaks associated with human morbidity and mortality [10]. During the recent years the number of WNV sequences, especially those of whole viral genome, is increasing, providing new insights and knowledge about the epidemiology and evolution of the virus [43,49].

### 3.2. Epidemiology of WNV in Humans

#### 3.2.1. Worldwide WNV Epidemiology

WNV was isolated for the first time from a febrile woman in 1937 in Uganda [50], while subsequent isolations took place some years later in Egypt [51] and then in Africa, Europe and Asia [52,53]. The first reported epidemic came from Israel in 1951 [54] with 123 cases of non-neuroinvasive disease. Cases were also reported the following year, while neuroinvasive cases (WNND, West Nile Neuroinvasive Disease) were recorded in 1957 and 1962 [54]. In Egypt, epidemiological investigations and serosurveys brought up to light high WNV endemicity in southern parts of the Nile delta in contrast to a low one in the parts neighboring to the Mediterranean coast [53]. In summer 1962, a WNV outbreak was recorded in France with several human cases of encephalitis, while two years later 13 human cases were reported and the virus was isolated from the blood of two entomologists as well [55]. A five-year seroepidemiological survey that was conducted in the region of the first reported cases, showed an antibody response of 4.9% [55]. More recent outbreaks were reported in some parts of Africa; in South Africa, six and five cases were recorded in 1974 [56] and 1983–1984 [57] respectively. During summer 1994, a total number of 50 cases was recorded in Algeria [58] including 20 WNND cases and one death. Two years later, during summer 2006, a human case of WNV encephalitis was recorded, but it is worth mentioning that 94 horses were found to be infected and 42 of them finally died [59]. Although serosurveys had shown a WNV activity since the 1960s in Romania, it was not until 1996 that a large-scale epidemic took place in Bucharest; national surveillance identified 393 hospitalized cases but the true number of cases could not be estimated; 17 deaths were recorded in people over 50 years old [5]. One year later, an epidemic took place during September-December with 173 WNND cases and eight deaths [55]. In the Volgograd region (Russia), 826 patients showed a clinical picture resembling that of WNND, but finally 183 was the number of confirmed WNV cases and 40 deaths were recorded [60]. In North America, the first emergence of the disease took place in New York City with 62 cases and seven deaths during the summer of 1999 [59,61,62], and it was soon spread across other states in the following years [59]; in 2000 to New Jersey and Connecticut, in 2001 to Florida, Louisiana, Maryland and Massachusetts and in 2002 to almost all states, reaching its peak of 9.862 cases in 2003 [63]. The largest state concerning population, California, had only sporadic cases in 2002–2003 but a large outbreak took place in 2004 with 778 cases and 28 deaths [64]. Other large-scale epidemics took place in Illinois (884 cases, 2002), Colorado (2,947 cases, 2003), Nebraska (1,942 cases, 2003), South Dakota (1,039 cases, 2003) and Texas (1,868 cases, 2012) [63]. In Canada, the first epidemic took place in 2002 with a total of 414 cases [65]. Circulation of the virus in countries of South America has been demonstrated through serological investigations, but human cases records remain only sporadic [1]. Meanwhile, several outbreaks had taken place in the Mediterranean Basin: in Israel (2000) [66], Russia (2000–2001) [59], Hungary (2008–2009) [67] and Italy (2008–2009) [68]. In Asia, outbreaks occasionally occurred in southern regions and especially in India, while sporadic cases were reported in Southeast Asia [69]. In Australia, Kunjin virus, considered to belong to a sublineage of WNV [70], has caused a total of 13 human cases during the period from 1992 to 2010 [71]. 

**Table 1:** The most significant WNV human outbreaks worldwide, 1950–2010.

Year	Country/State	Cases	Deaths	Reference
1951	Israel	123	0	[54]
1962	France	Several encephalitis cases		[55]
1964	France	15	1	[55]
1974	South Africa	6		[56]
1983–1984	South Africa	5		[57]
1994	Algeria	50	8	[58]
1996	Morocco	1	1	[59]
1996	Romania	393	17	[5,72]
1997	Tunisia	173	8	[55]
1999	Russia	183	40	[60]
1999	New York State	62	7	[62]
2000	Israel	428	42	[66]
2000–2001	Russia	120		[59]
2002	Canada	414		[65]
2004	California	778	28	[64]
2008	Hungary	22		[67]
2009	Hungary	9		[67]
2008–2009	Italy	26		[68]
2010	Greece	262	35	[7]

**Figure 2:**
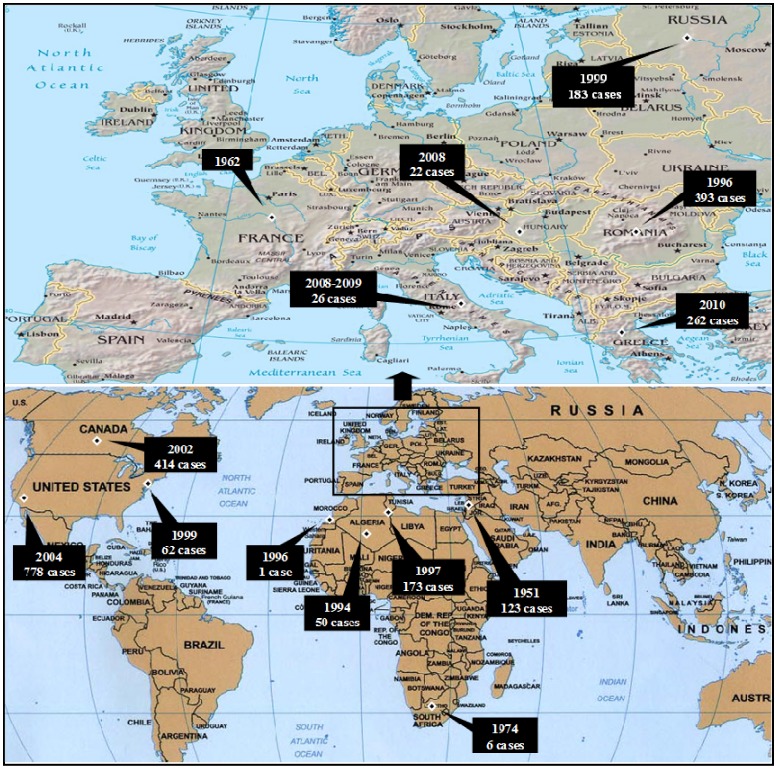
The most significant WNV human outbreaks worldwide, 1950–2010.

**Figure 3:**
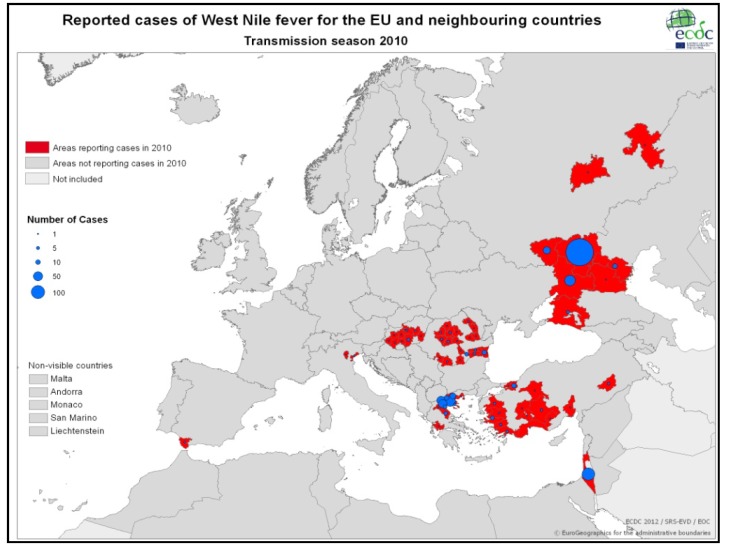
WNV human cases in EU and neighboring countries, 2010 (Source: http://www.ecdc.europa.eu/).

**Figure 4:**
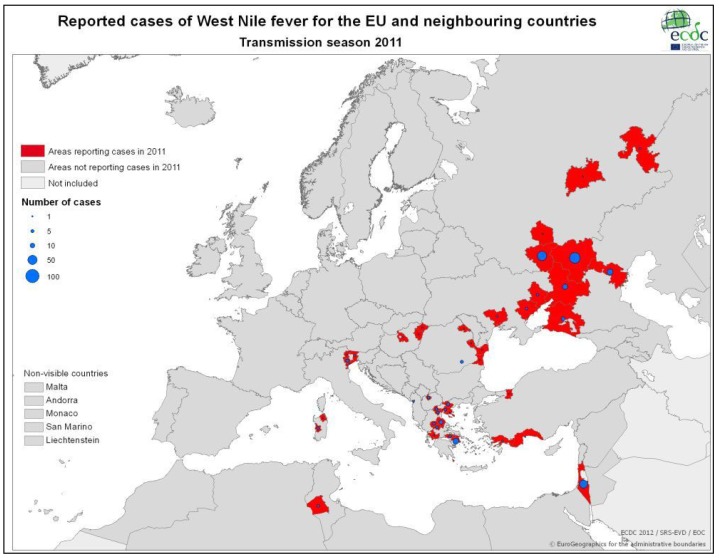
WNV human cases in EU and neighboring countries, 2011 (Source: http://www.ecdc.europa.eu/).

**Figure 5:**
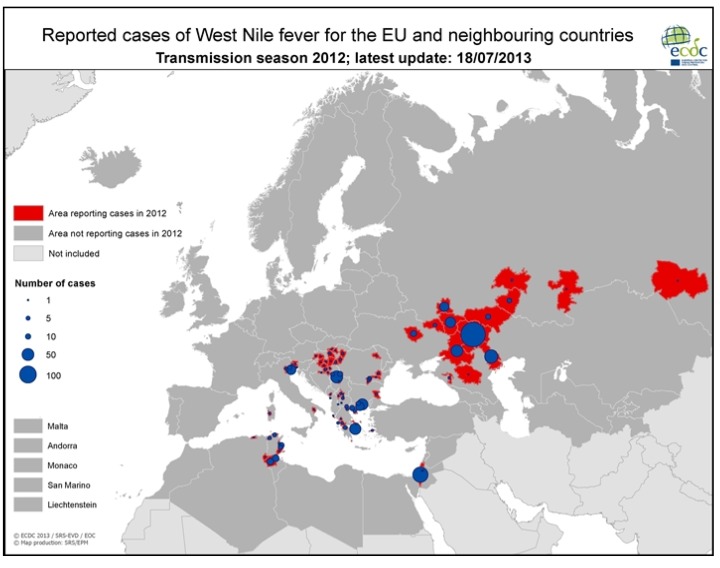
WNV human cases in EU and neighboring countries, 2012 (Source: http://www.ecdc.europa.eu/).

Table 1 and Figure 2 demonstrate the most significant outbreaks worldwide during the sixty-year-period of 1950–2010 with an emphasis on the Mediterranean Basin. According to ECDC data, in 2010 WNV human cases were detected in several European Union (EU) countries (Figure 3) such as Greece [73], Austria, Romania, Hungary, Italy and Spain, while outbreaks occurred in Russia and in Turkey as well. In Romania, a total number of 170 suspected cases were reported; 57 were cofirmed cases (54 with West Nile neuroinvasive disease-WNND and three with fever), while five deaths were recorded in elder people with underlying conditions [74]. In Italy, for the third year in a row, WNV human cases were identified; all six cases (three WNND and three with fever) were located further north in relation to previous virus activity [75]. Meanwhile in Spain, although seropositivity for humans had been reported since 2001, the first WNV cases were recorded during autumn 2010 [76]. Thirty-four probable cases along with 12 confirmed cases were reported for the first time in Turkey during 2010; of the 47 cases, 10 died [77]. In 2011, 128 cases were reported in EU member countries (100 cases in Greece, 14 in Italy, 11 in Romania, three in Hungary) and 212 in neighbouring countries (Albania, FYROM, Israel, Russian Federation, Tunisia, Turkey and Ukraine), as shown in Figure 4. During 2012 transmission season, 242 cases were recorded in EU countries (161 in Greece, 50 in Italy, 17 in Hungary and 14 in Romania) and 693 in neighbouring countries (Algeria, Croatia, FYROM, Israel, Kosovo, Montenegro, Occupied Palestinian territory, Russian Federation, Serbia, Tunisia and Ukraine), as depicted in Figure 5.

#### 3.2.2. Epidemiology of WNV in Greece

Although WNV infections in humans and equines had already been reported since the 1950s in Mediterranean countries, no cases were ever reported in Greece. Nevertheless, serological studies have demonstrated WNV antibody prevalence in animals and humans during the 1970s and 1980s [78], while a study using hemaglutination test demonstrated a 8.6% seroprevalence in samples of serum collected during 1963–1964 [79,80]. A study conducted to find out what other arboviruses occur in Greece, showed a seroprevalence of 1.15% (13 positive out of 1,128 sera samples) especially in Athens, Peloponnese, Epirus and Aegean islands [81]. Also, a survey using Plaque Reduction Neutralization (PRN) and Immunofluorescence Assay (IFA) in 1990 showed that WNV or an antigenically related flavivirus was present and active in Greece, as ~1% of the human population (residents of Imathia and Lakonia districts) carried WNV IgG antibodies [82]. During the decade 2000–2009, all encephalitis cases that were tested in the National Reference Center for Arboviruses were negative for WNV. In order to estimate the seroprevalence of WNV IgG antibodies in residents of northern Greece prior to the 2010 epidemic, 626 stored samples from the two-year period 2003–2004 were examined; the level of seroprevalence was 0.62% [83]. Blood donors’ samples (n = 9,590) from seven Greek hospitals and samples obtained from patients with aseptic encephalitis (n = 115), collected from the National School of Public Health and the University Hospital of Ioannina (West Greece) were examined by a Nucleic Acid testing (NAT) during a study from 2006 to 2007; the study aimed to detect the presence of the virus but all samples turned out negative [84]. The most recent seroprevalence study prior to the outbreaks took place in 2007 in Imathia district (district of North Greece that neighbors three different rivers) under the coordination of Aristotle University of Thessaloniki. During spring 2007, 392 serum samples were collected from persons who visited Alexandria Health Center (Alexandria is a city of Imathia district). Enzyme-Linked Immunosorbent Assay (ELISA) and IFA were used in order to detect WNV IgG antibodies; six were positive and four of them had neutralizing IgG antibodies against WNV [79]. It is worth mentioning that Alexandria was the epicenter of 2010 epidemic [85].

**Figure 6:**
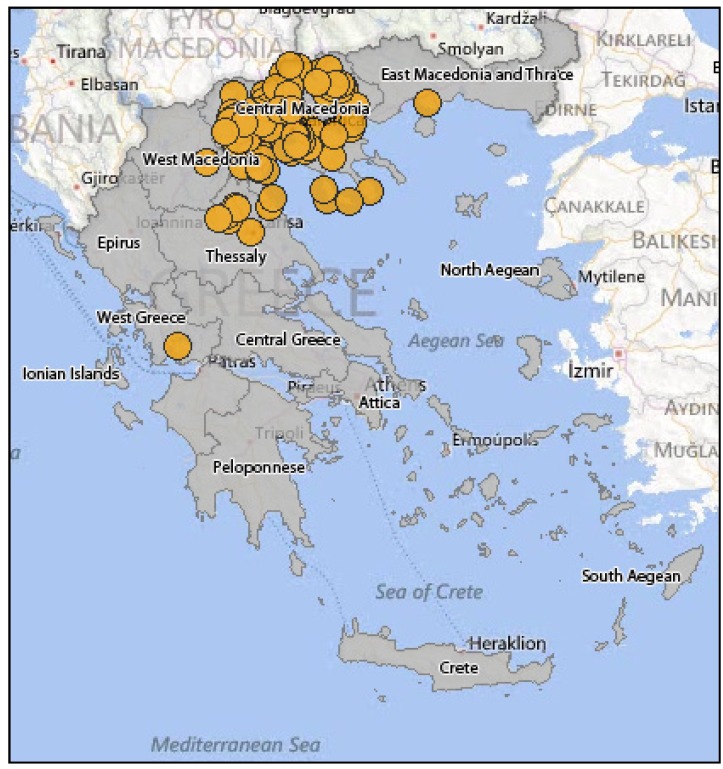
WNV human cases in Greece, 2010.

**Figure 7:**
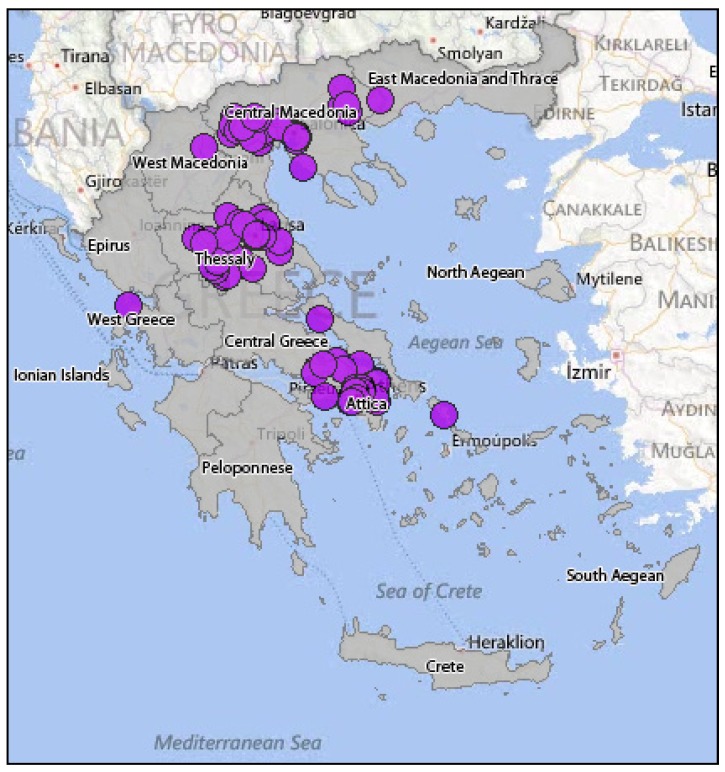
WNV human cases in Greece, 2011.

**Figure 8:**
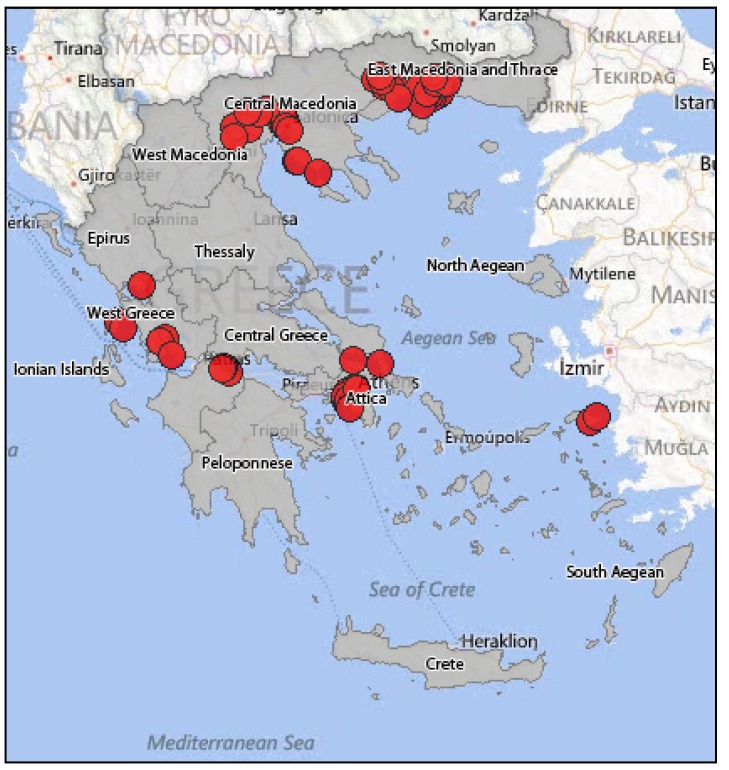
WNV human cases in Greece, 2012.

In summer 2010, human WNV infections were detected in Greece for the first time. Until November 2010, 262 cases were reported (Figure 6); 197 of them displayed a WNV neuroinvasive disease (75%), while 65 were non-neuroinvasive cases with mild symptoms (25%). The largest number of WNND was recorded in Central Macedonia (districts of Imathia, Kilkis, Thessaloniki, Serres) [86]. The mean age of infection was 72 years, while the 109 cases (55%) were male and 88 (45%) were female. The case fatality rate of the WNND cases was 17.7%, while a total of 35 deaths were recorded in elderly people who had underlying disease [86]. Especially among WNND cases, 168 (85%) showed symptoms of encephalitis or meningoencephalitis, 23 (12%) had an aseptic meningitis clinical picture, while six (3%) showed only acute flaccid paralysis signs [86]. In early summer 2011, one month after the seroconversion was observed in sentinel chicken [47], the first human WNV case was diagnosed, indicating the onset of the 2011 epidemic (Figure 7). That year, Thessaly (Central Greece) was more affected than Central Macedonia (North Greece), which was the epicenter of 2010 outbreak, while human cases were recorded also in East Attica (South Greece), where no cases had been reported in 2010 [87]. The total number of cases that were reported was 100 (not including an imported case from Albania), out of which 75 were WNND and 25 with mild symptoms, while the case fatality rate for the WNND was 12% with nine deaths observed in persons over the age of 65 [88]. It is worth mentioning that one of the WNND cases was a French national that was diagnosed in France but had stayed in Greece throughout the incubation period [88]. During the 2012 transmission season, 161 cases were recorded in total, not including two imported cases from the USA. One hundred and nine (109) cases were WNND, including 18 deaths, while the rest 52 were cases with mild symptoms (mainly fever) [89]. The greater incidence levels were recorded in Attica region and in North Greece. One of the WNND cases was a German national that was diagnosed in Germany but infected in Greece [89]. Figure 8 shows the WNV human cases in Greece during 2012.

### 3.3. Clinical Course

The incubation period for WNV infection is 2–15 days [90]. Most of the infections (80%) are asymptomatic, while 20% are presented as mild febrile syndrome experiencing headache, myalgias and arthralgias, nausea and vomiting [2] with or without exanthema (on arms, legs or trunk) [91] usually in younger patients [92], often accompanied by lymphadenopathy. This form of the disease, known as West Nile fever (WNF), lasts generally less than one week, but some patients may complain about a severe sense of lassitude and malaise during recovery [2]. A follow-up study in WNF patients demonstrated that fatigue and weakness lasted for almost a month since the onset of the symptoms [91]. A study among Greek patients with WNF showed that many of the patients presented persistence of fever, intense malaise or even experienced weight loss [93]. A long-lasting recovery of several days or even months, suggest that WNF is not always mild [93]. Approximately 1% of the patients with WNV infection present symptoms from the nervous system; primary target of WNV in the brain and spinal cord are the neurons. Most of these WNND cases are classified in three syndromes: West Nile meningitis, West Nile encephalitis and acute flaccid paralysis (poliomyelitis-like syndrome); it is worth mentioning that features of these syndromes may overlap in the same patient [94]. West Nile meningitis displays high fever and signs of meningeal irritation, such as severe headache, photophobia and nuchal rigidity [95], while Kernig’s and Brudzinski’s signs may be found during the physical examination of the patient [94]. West Nile encephalitis is characterized by sudden and high fever, severe headache, photophobia, altered consciousness level (e.g., disorientation or even coma) and mental status, or even focal neurological signs, such as ataxia, seizures or dysarthria [4,94]. There have been several reports of dyskinesias or even parkinsonian features accompanying West Nile encephalitis [96]. Although not usual, some other syndromes have been also reported, such as rhabdomyolisis, myositis [97] and autonomous nervous system involvement [98]. Acute flaccid paralysis occurs in approximately one out of ten patients with WNND [95] and includes asymmetric limb weakness, hyporeflexia and sensor abnormalities. Most of the times, the clinical picture reminds us of a polio-like syndrome of assymetrical paralysis, while only in a few cases, it may present a Guillain-Barré-like syndrome with a respiratory muscles paralysis [92]. Neuro-invasive disease is more common among immunocompromised and elders. It has been shown that advanced age remains the most important risk factor for severe neurologic infection [90]. 

Following the bite of a WNV-infected mosquito, the infected skin Langerhans dendritic cells migrate to the lymph nodes, where initial replication takes place; thereafter the virus is spread through the blood stream to visceral organs, such as the kidney and the spleen, where a second replication occurs [99]. Host innate and adaptive immunity mechanisms are initiated in an effort to limit WNV replication, neuroinvasion and dissemination in the CNS and to prevent neuronal injury; type I interferons and pro-inflammatory cytokines act as antiviral agents [100]. IgM and IgG antibodies are developed rapidly after viremia and before WNV-RNA levels become undetectable in the bloodstream [101]. WNV crosses the blood-brain barrier via multiple mechanisms including: (1) the “Trojan horse” model (intracellular transport within macrophages or neutrophils), (2) loss of integrity of the blood-brain barrier, cytokine-mediated (TNF-α, MIF) or matrix metalloproteinases disruption of tight junctions and basement membranes, (3) direct infection of brain microvascular endothelial cells with basolateral spread of the virus, (4) infection of choroid plexus epithelial cells, or (5) direct infection of olfactory neurons adjacent to the cribriform plate [102]. Various mechanisms (e.g., activation of CD8+ T cells, granzyme-dependent granule exocytosis pathway, apoptosis) play a role in the WNV clearance in the CNS [102]. Knowledge of the mechanisms of the virus entry to the CNS and its clearance will enable design of proper treatment strategies. 

Abnormal magnetic resonance imaging findings in patients with WNV meningoencephalomyelitis are nonspecific but not uncommon. Anatomic areas commonly affected are basal ganglia, thalami, mesial temporal structures, brain stem, and cerebellum [103]. Cerebrospinal fluid (CSF) examination generally shows lymphocytic pleocytosis, but neutrophils may predominate early in the course of illness [104]. The pathological findings are typical of a viral meningoencephalitis and include microglial nodules, perivascular chronic inflammation, and variable neuronal loss with necrosis or neuronophagia [3].

Complications are often seen in patients with WNND, and include malaise, tremor, headache, amnesia, and depression, while several ocular complications, such as optic neuritis, choriorenitis and retinal heamorrhages, have been reported as well [96]. A follow-up study in 22 WNND survivors (20 patients with encephalitis, two with meningoencephalitis) in Greece, where WNV lineage 2 caused large outbreaks for three consecutive years (2010-2012), showed that the most common symptoms remaining after one year of infection were anorexia (77.3%) and muscle weakness (72.7%), followed by memory impairment (36.4%) and depression (22.7%), while older age was correlated with memory impairment, muscle weakness, and permanent neurological damage [105].

### 3.4. Laboratory Diagnosis

The laboratory diagnosis is mainly based on the detection of WNV-specific IgM and increasing titres of IgG antibodies in serum and/or CSF. When anti-WNV IgM antibodies are detected in CSF, the case is considered as confirmed WNV infection [101]. Both ELISA and IFA can be used in serological examination. It is worth mentioning that during the WNV outbreak in 2010 in Greece, it was observed that although IgM and IgG antibodies were present early in the WNND cases, a delay in antibody production was observed in the non-neuroinvasive cases [106]. On the other hand, persistence of WNV IgM antibodies (sometimes for more than one year) is also a common finding in WNV infections. During a 2010 study in Greece, a second serum sample was taken from 29 WNV infected patients 75–180 days after symptoms onset and a third sample was taken from eight out of the 12 patients that had a positive for IgM second sample. The study showed that 12% of patients had persistent IgM presence at 181–270 days of follow-up [106]. Persistence of IgM antibodies may cause problems in discriminating an acute from a previous infection; avidity tests usually enable the discrimination, and WNV IgG avidity index <40% (together with the presence of IgM antibodies) supports the presumption of recent infection (<20 days) [107,108,109]. Is should be always kept in mind that cross-reactions in serology are common among flaviviruses [92], and caution is needed for the interpretation of serological results of flaviviral infections. Efforts have to be focused on the development of novel diagnostic assays with increased specificity [110]. Plaque reduction neutralization test (PRNT) is used for differentiation of the antibodies produced against different flaviviruses, but it is a time-consuming examination that is usually performed only in reference laboratories. Best results are obtained when convalescent sera are tested. Moreover, some degree of cross*-*reaction in PRNT may cause ambiguous results. A detailed travel and vaccination history (previous vaccination against Yellow fever and Japanese encephalitis viruses) is essential for WNV serology results interpretation. 

Nucleic acid testing (ΝΑΤ) is the method of choice for the screening of blood donors. However, polymerase chain reaction (PCR) is not very helpful for the diagnosis of WNV infections due to low and short viremia in patients. Studies in animal models showed that WNV is shed for a longer time in the urine of WNV patients [111], and recent reports showed that WNV RNA is detectable in urine at a higher rate and load and for longer time than in blood; therefore, urine might be better sample for the WNV molecular diagnostics [44,48,112]. Virus isolation is rarely used for diagnostic purposes; it is performed in reference laboratories and it is of limited use because of the short duration of viremia and the low titer of the virus in the bloodstream of WNV-infected patients. However, WNV is easily isolated in cell cultures from NAT-positive blood donors [45,113] and from urine of patients with WNV infection [44]. 

In conclusion, the best method for laboratory diagnosis of WNV infections is the application of serological methods accompanied by confirmatory examination and combined with molecular methods performed preferably on urine samples when needed. 

### 3.5. Treatment

There is no specific treatment available for WNV infection and those with mild symptoms do not need any special care. Severe cases are provided with supportive care; in WNND cases, hospitalization is required, while critically ill patients may need special management in an intensive care unit. Encephalitis cases, in particular, should be constantly monitored for elevated intracranial pressure or seizures, while special attention should be given in cases that may need respiratory support [97].

Interferon-a and ribavirin, usually used for the treatment of viral infections, have been tried as specific WNV treatments but only *in vitro*, as no clinical trials have been completed [114]. It has been hypothesized that one of the modes of action for ribavirin against WNV is the error-prone replication [115]. Drugs that can alter the cascade of immunobiochemical events leading to neuronal death may be potentially useful (high-dose corticosteroids, interferon preparations and immunoglobulin containing WNV-specific antibodies) [116]. Intravenous administration of human immunoglobulin seems to be effective for the treatment of WNV infection [117,118]; the prophylactic and therapeutic efficacy examination gave encouraging results in mice [117], while the immediate administration to a suspected case, despite the initial negative serology test, led to quick recovery of a West Nile virus encephalitis case [118]. Toll-like receptor agonists and drugs that inhibit specific cytokines, as well as interferon preparations, have shown potential therapeutic efficacy, while humanized monoclonal antibodies directed against specific viral proteins have been developed and evaluated in patients with WNV infection [119].

### 3.6. Human Vaccines

Despite intensive efforts towards the development of effective WNV prevention during the last decade, no licensed human vaccine is available. The fact that WNV belongs to a certain genus, having common characteristics with viruses, such as dengue virus, Yellow fever virus, Japanese encephalitis virus, enables scientists to construct a candidate based on previous vaccine discoveries that have managed to control the spread of the abovementioned pathogens. Up to know research has offered several vaccine candidates.

Just after the first epidemic in the USA during 1999, the National Institute of Allergy and Infectious Diseases (NIAID) started to work on a “chimeric” WNV/Yellow fever vaccine named ChimeriVax-WN02 [120]. It is a live attenuated vaccine which is based on the licensed Yellow fever 17D vaccine virus. Initially, the premembrane (PrM) and envelope (E) genes of Yellow fever were replaced by those of the WNV NY99 strain forming the Chimeri-Vax-WN01 that has been used in animals; further attenuation by mutations in E protein led to the ChimeriVax-WN02 construction [121]. The preclinical studies on hamsters, mice, monkeys and horses were successful, followed by promising results in 18-40-year-populations in Phase I clinical trial [120,122,123]. Phase II clinical trial showed high immunogenicity and well-tolerance in both parts of the study [120,124]; the first part included individuals aged 18–40 years old, while the second part included subjects aged 41–64 years old and people over 65 years old [124]. Based on dengue virus, scientists constructed a live attenuated chimeric vaccine that combines genes from WNV and dengue virus 4 (WN/DEN4-3’delta30), expressing again WNV PrM and E proteins [125]. Successful preclinical trials in mice and monkeys led to Phase I clinical trial that is taking place now. The VRC is a 3-dose DNA vaccine which encodes PrM and E glycoproteins under a cytomegalovirus-T-cell leukemia virus type I R region promoter (CMV/R) [121]. It has gone under two Phase I clinical trials where safety, tolerability and adequate immunogenicity were shown in groups of 18-to-50-years-old and 51-to-65-years-old subjects [126,127]. A *Drosophila*-expressed subunit vaccine candidate, named HBV-002 (also known as WN-80E), has been constructed based on the E protein (without the transmembrane domain); it has completed a successful Phase I clinical trial, showing adequate safety and immunogenicity levels [121]. Some other candidates, that are now in preclinical development, include: a recombinant subunit vaccine based on soluble protein E produced in *E. coli* which showed satisfactory results when administered to mice [128], Baculovirus-produced subunit vaccine which induced antibody response in mice [120], candidates based on EDIII (E protein Domain III) [129], pKUNdC/C which is a vaccine based on Kunjin virus (WNV strain in Australia) [120,130], a candidate named RepliVAX which is a non-replicating vaccine [131] that demonstrated protective immune response in mice, hamsters and non-human primates. At the same time, many projects of inactivated vaccines, WNV protein vaccines and synthetic-peptide-based vaccines are also under evaluation [120]. Although there is a lack of commercial interest for a human WNV vaccine [129], a number of vaccine candidates are currently being tested that seem promising. A comprehensive table that includes the candidates that are currently under clinical development is provided below (Table 2).

**Table 2:** West Nile virus vaccine candidates under clinical development.

Vaccine Cadidate	Antigen	Clinical Trial Phase
ChimeriVax-WN02	Yellow Fever 17D expressing WNV PrM/E	II
WN/DEN4-3’delta30	Dengue virus 4 expressing WNV PrM/E	I
VRC	DNA expressing PrM/E	I
HBV-002	Soluble E protein (no membrane domain)	I

### 3.7. Non-Vector-Borne Transmission

Even though the main mode of transmission is via infected mosquito bite, it has been shown that alternative less common modes of WNV transmission also exist: (1) through transfusion of infected blood and blood products, (2) through solid organ transplantation from an infected donor to a healthy recipient, (3) through the placenta from an infected mother to her fetus (vertical transmission) and (4) through occupational infection concerning mainly laboratory professionals. Virus transmission is possible through transfusion of red blood cells, platelets and fresh-frozen plasma [132] with the first cases being reported in 2002 [133]. In 2003, based on this incident, the blood-collection agencies around USA proceeded to screening of six million blood units with NAT test resulting in the removal of 818 positive for the virus units [134]. Routine testing of the American Red Cross during 2003–2004 identified 540 donations that were WNV RNA positive; 367 of them were IgM antibody negative and thus infectious [135]. Although this technique is the one widely used for blood unit examination, a case of transmission despite the NAT negative result was reported in a Nebraska man that had received transfusion after surgery and presented with encephalitis after 13 days [136], followed by a failure of NAT to detect units with a low viremia level when examined in minipools [101]. A more targeted (individual-donation) NAT rather than the usual minipool examination has been suggested [137] especially in regions with already known high WNV incidence levels, while a lot of progress has been done for more rapid and accurate assays *in vitro* [138]. Meanwhile, during 2002, a risk assessment that took place in specific metropolitan areas of the USA with high-incidence of WNV infections estimated a mean risk for WNV transmission through blood products ranging from 1.46 to 12.33 per 10,000 donations [139]. Three years after the introduction of the virus in the USA, transmission through solid organ transplantation was first reported in 2002 [140,141] followed by five additional clusters the following years [142]. It is worth mentioning that the investigation that followed the infection of four organ recipients from an infected donor, demonstrated that the donor had been probably infected through blood transfusion [140]. Currently, there is not any national policy that requires organ donors screening, but serious cases of neuroinvasive disease in recipients implies a need for ELISA and NAT testing of donors during transmission season [143]. In 2002; a 20-year-old WNV infected woman delivered at term a live infant with chorioretinits and severe cerebral abnormalities (white matter loss, focal cerebral destruction) that was positive for WNV-specific IgM and neutralizing antibodies [144]. This was the first report of transplacental WNV transmission in humans. It was followed by cases of major birth defects (polydactyly, microcephaly, lissencephaly) in newborns whose mothers’ were infected, but according to the study conducted during 2003–2004, it was not clear whether some of the abnormalities of this cohort could be attributed to congenital WNV transmission [145]. One case of probable WNV transmission through breast milk was reported in 2002 [146] but since there was no confirmed case reported from that time, transmission via breast-feeding seems to be an unlikely way of non-vector-borne transmission of WNV [61,147,148]. Two cases of laboratory-acquired infection were reported in the USA; the most probable mode of transmission was through percutaneous inoculation [61,149,150] or even through exposure to aerosol [61], as shown previously in mice [151]. Finally, in Wisconsin, during 2002, two turkey breeders deployed a WNV clinical picture. The investigation that followed showed that the workers as well as the turkeys they were handling were WNV infected; nonetheless, the mode of transmission to these workers remains unknown [152].

### 3.8. Preventive Measures for Humans

Since there is no available vaccine for human protection from WNV infection, appropriate preventive measures should be taken. People should be informed about the peak of the mosquito activity hours (from dusk till dawn) so that they avoid being outdoors during these hours and use insect repellents (e.g., DEET, picaridin, IR3535), that should be applied on uncovered body parts and even on clothes. Protective clothing (long sleeves, long pants, socks), if possible, is an additional effective measure against mosquito biting. As for domestic protective measures, screens in windows and doors and nets (especially for small children) should be used, while elimination of backwater is also necessary. People should empty water from flower pots, bird baths, buckets and cans, get rid of discarded tires that can collect water and favor mosquito growth, and clean clogged rain gutters. In addition, the use of air-conditioner or fan seems to be helpful as the cold streams prevents mosquitoes from approaching. Although it has not been proved that handling sick animals may lead to WNV transmission, CDC recommends people who handle tissues of sick animals or work in slaughtering and culling sections to wear gloves and accompanying protective clothes [153]. Laboratory workers should take the regular precautions and try not to over-produce aerosols [150]. Pregnant women should protect themselves from mosquito bites and should undergo diagnostic testing when necessary [144], while the health benefits of breast-feeding and the fact that this mode of transmission seems rather not possible, there is no regulation suggesting breast-feeding stopping [146]. Finally, laboratory testing should be implemented on blood units, especially in affected regions. Polymerase chain reaction (NAT technology) is generally used for blood units testing, while no policy for organ donors testing has been established so far [142]. Table 3 summarizes the protective methods that should be followed, divided according to the modes of transmission that were previously described.

**Table 3:** Preventive measures for humans against WNV.

Mode of Transmission	Protective Measures
Mosquito bite	Personal protective measures
	Avoiding being outdoors during mosquito peak activity hours
	Use of insect repellents
	Use of protecting clothes
	Domestic protective measures
	Use of screens
	Use of bed nets
	Use of air-conditioner/fan
	Elimination of backwater
Animal-to-human	Use of gloves
	Use of protective clothes
Laboratory-acquired infection	Standard contact and droplet precautions
	Minimizing aerosol production
Intrauterine	Risk infection reduction by taking protective measures throughout pregnancy
	Diagnostic testing when clinically appropriate
Breastfeeding	Breastfeeding recommendations have not changed
Transfusion	NAT technology for blood units testing
Transplantation	There is no policy for organ donors screening

### 3.9. Human Epidemiological Surveillance

The general goal of human surveillance for WNV is to prevent severe complications and deaths linked to the virus. Specific objectives of surveillance are the disease impact assessment on public health, the monitoring of disease trend and distribution, the intervention activities evaluation and the identification of risk factors and high-risk populations [154].

According to the Hellenic Center for Disease Control and Prevention (HCDCP), a patient is considered WNV infected when they meet one of the clinical AND one of the laboratory criteria, while case classification into probable or confirmed case is conducted by the HCDCP. Both clinical and laboratory criteria are presented in Table 4 below.

**Table 4:** Clinical and laboratory criteria for WNV case definition.

Type of Criteria	Analysis of Criteria
Clinical	Encephalitis (disturbance of consciousness and fever) OR Aseptic meningitis OR Other acute clinical manifestations from the CNS or the peripheral nervous system (paresis, sensory disturbances, *etc*.) OR Fever without clinical manifestations from the nervous system and absence of any other possible diagnosis.
Laboratory	Virus isolation/detection in blood or CSF or other tissue OR Detection of WNV-specific IgM antibodies in CSF OR High titers of IgM antibodies or increasing IgG antibody titer in consecutive serum samples.

WNV infection should be included in the National Mandatory System. Healthcare professionals should be familiar with the case report form and the disease recording system of their country. The information that every clinician should be aware of concerning specimen sending and case reporting is provided in Table 5 that follows.

**Table 5:** Specimen sending for WNV diagnosis and case reporting instructions.

Procedure	Details
Case reporting form	The fields that should be filled are: demographics, risk factors (occupation, recent trip abroad, recent move to another part of the country, blood transfusion, *etc*.), clinical features (symptoms, date of onset, symptoms from the nervous system *etc*.), laboratory results and the personal details of the attending physician
Clinical specimens	Specimens of serum (minimum quantity: 2 mL) and CSF (minimum quantity: 0.5 mL) should be transported to the Reference Center in special vials in a special container for biological samples. All samples should be labeled with patient’s personal details, sampling date and the hospital responsible for sampling. The accompanying consignment note is prerequisite
Consignment note	Information that should be included is: clinical features of the patient, sample type (serum, CSF) and personal details of the attending physician

Human surveillance is divided in two major categories that are presented in Table 6 accompanied by detailed explanation of each type directions. Enhanced passive surveillance is implemented on hospitalized cases of encephalitis and WNV IgM positive patients in regions with unknown virus activity; therefore it concerns mainly primary health care providers, infectious disease physicians and neurologists. On the contrary, active surveillance should be implemented in known affected areas.

**Table 6:** Types of WNV human surveillance.

Surveillance Type	Directions
Enhanced passive surveillance	Clinical suspicion of WNV infection should not be underestimated, particularly during the season of high mosquito activity (June to October) [68]. In case of diagnostic doubts, a stand-by accredited laboratory should be available for confirmation and the specimens should be sent as soon as possible. CSF samples should be collected in pairs: one sample during acute disease (0–8 days) and one sample during convalescent disease (14–21 days) in order to observe seroconversion as well as for increased accuracy of the serological testing [154].
Active surveillance	Regular contact with physicians and hospital public health workers asking about suspected cases is necessary. Thus, diagnosis can be improved together with reporting of the disease. Laboratory surveillance should be implemented for non-specific results of CSF serological testing [154].

### 3.10. WNV Risk Assessment

#### 3.10.1. Introduction

Since 1999, when the first WNV cases appeared in the USA, technological and model spatial patterns improvements offer the opportunity for risk assessment based on dynamic space-time data illustrations. Mapping technologies are used to associate the WNV risk with habitat and virus activity characteristics. Early warning systems for WNV outbreaks can provide a basis for surveillance and targeted control interventions.

#### 3.10.2. The Use of Geographic Information Systems (GIS) in the Risk Assessment of WNV

The study of various diseases in relation to space is a complex science that has relied heavily on the potential of technological developments. An example of the above procedure is GIS or Systems of Geographic Information. 

GIS was mainly developed in the 80s, offering not only high data processing and analysis, but also accuracy [155]. A GIS is an organized collection of computer systems engineering (hardware), software systems (software), spatial data and human resources to collect, record, gather data, manage and analyze information in any form relating to the geographical environment [156]. For several years in many countries GIS and remote sensing are applied on the study of diseases and their association with environmental and socio-economic parameters. 

The spatial analysis is the focus of GIS systems because it includes all transformations, manipulations and methods that can be applied to geographic data in order to support decision making. Queries are the most basic analysis mode, in which the GIS system is used to respond to user needs. Examples of queries regarding issues of disease and GIS are: [157]

- Where cases of disease A are located?
- Where spread of WNV to humans is located in relation to vectors of the virus?
- How is the spread of the virus correlated with environmental factors such as altitude, land use, distance from water surfaces?
- How is the spread of the virus correlated with demographic or socio-economic factors such as age, sex, occupation, living conditions, income?
- Will spread of the virus be different in the future if environmental conditions remain the same?
- Which is the number of cases in each area of ​​interest in proportion to the population over the age of 60 years?
- May the spatial distribution of the disease A be due to a similar distribution of population sizes?
- Which is the range of antibody levels for disease A in people’s blood? Are they randomly distributed in the area of the city or have distinct geographical patterns?
- How are any patterns correlated with the characteristics of living conditions?

Spatial models provide scientists with spatial analysis tools for an “extension” of measurements of field data and correlation-integration complex information in space and time. The models generated by the investigator or study team should be applicable in practice, and should also be accurate, durable and effective in relation to the cost of implementation (accurate, robust, cost-effective). The best evaluation is done with a control model that uses new data from the population or sample of the original model [158]. The model evaluation is particularly important in understanding the accuracy of predictions [159]. The effectiveness of a model can be achieved if the accuracy of model predictions is calculated [158]. In order to develop a model of spreading, the use of two independent samples is proposed, one for setting (calibration) and the other for the evaluation of the model [160].

In 1999 the appearance of WNV in New York was the first time the disease was recorded in the Western Hemisphere. From 1999 onwards, the virus spread to other states such as New Jersey, Connecticut, Massachusetts, and Maryland, forcing authorities to intensify their surveillance efforts by significantly developing GIS [161]. In a recent study in Canada, GIS, mosquito population data, climate data and land use were combined to identify areas of high probability of WNV dispersion [162]. In the state of Mississippi GIS in conjunction with data of birds positive to the virus, bioclimatic indices, vegetation and stream network used in the creation of many spatial models to identify and control the virus in spatial scale of municipalities and regions. The system also combined data from the breeding areas of mosquitoes and dead of wild birds while in order to verify the models data from human cases were used [163].

The use of logistic regression in conjunction with the development of GIS has increased in research relating to WNV in recent years [164,165,166,167,168]. Logistic regression is a method of multivariate statistical analysis that uses a set of independent variables to investigate the variability of a categorical dependent variable. Data used in the analysis are not necessary to follow a normal distribution.

The prediction is based on the construction of a linear model, namely the determination of the values of the coefficients of a set of independent variables used as predictor variables. Besides prediction, a logistic regression model enables scientists to estimate the effect of each independent variable in the value of the dependent variable. As the mean value of the continuous variable Y for a given set of values of the independent variables is estimated, with the aid of the linear regression model the probability of success (p) of a categorical variable value can be estimated for a set of one or more independent variables [169]. The method of logistic regression and GIS were used to develop spatial models to predict possible areas of exposure to WNV in the eastern and western Colorado in the USA [170]. Apart from the climatic data, the data of land use and topographic configuration used, the NDVI (Normalized Difference Vegetation Index) was also used. 

Since 2001 GIS applications and dissemination of data systems and internet applications (Web GIS) have been developed in Canada in order to meet the needs of the surveillance for WNV and it is worth mentioning that the operating costs are very high [171]. In a similar survey in Ohio, USA in early 2003, the GIS and spatial statistical methodology was used to identify the environmental and demographic factors that have a positive effect on the risk of virus transmission [172]. GIS combined with multivariate statistical techniques in addition to the cartography of areas with increased risk of disease allowed the determination of risk factors through a multitude of environmental and socio-economic parameters related to the ecology and epidemiology of WNV as socioeconomic conditions [168].

Finally, in Greece although GIS is an important tool for public health risk assessment and interventions and in particular for the management and prevention of diseases like WNV, it has not yet been sufficiently evaluated. Few studies have been performed with the application of GIS study on WNV [173,174]. It is worth mentioning that the monitoring and control programmes launched recently aim to develop the capabilities of GIS, and to contribute with useful information and data on the spatial dimension of the WNV and its outbreaks to the decision makers to enable them to make the better decisions and take operational actions creating prevention and control techniques [175].

#### 3.10.3. Recent Risk Assessment Models

The first early warning system for WNV was established in New York City from the Department of Health in 2000 when the first WNV outbreak took place. This system was based on dead crow surveillance. The Health Information Network (HIN) was the main component of this system. The HIN was used by local authorities to enter data about sick or dead birds. Then, dead crows were collected for laboratory testing and the results of virus-positive birds were used in the model. The bird surveillance data and virus positive results were used to create maps which predicted viral activity [176]. 

The systems used by the Department of Health in New York showed that the number of dead crows increases much faster than the number of infected birds or mosquitoes and that dead crow monitoring is much more efficient for outbreaks detection [177]. The use of mosquito-to-crow ratio, that followed, showed that, while mosquito control decreases the virus outbreak threshold, the bird control increases it [177].

The California State Mosquito-Borne Surveillance and Response Plan was initially developed for the surveillance and risk assessment of western equine encephalitis virus, St. Louis encephalitis virus and other arboviruses surveillance and risk assessment and has been used since 1969 [178]. After the first WNV cases in 1999 in the USA, the California Department of Health Services (DHS) revised the model in order to include WNV in the risk assessment procedure. According to this model, seven risk factors (Table 7) are evaluated in a five-value rank and their average score represents the overall risk. WNV activity is categorized into normal season (score: 1–2.5), emergency planning condition (score: 2.6–4) and epidemic condition (score: 4.1–5).

**Table 7:** Risk factors and thresholds for WNV risk assessment [179].

Risk Level	Rain and Temperature Levels	Mosquito Abundance	Mosquito MIR^*^/1,000	Chicken Sero- Conversions	Equine Cases	Human Cases	Proximity of WNV Activity to Residential Areas
1	Significantly below average	<50%	0	0	0 throughout state	0 throughout state	Remote area
2	Below average	50%–90%	0.1–1.0	1			Rural area
3	Average	91%–150%	1.1–2.0	PF^*^ > 1 C/PF < 1	>1 throughout state 0 local	<1 throughout state 0 local	Small town
4	Above Average	151%–300%	2.1–5.0	PF > 1 1 < C/PF ^*^ < 3	1–2 local		Suburban area
5	Significantly above average	>300%	>5.0	PF > 1 C/PF > 3	>2 local	>1 local	Urban area

Notes: * MIR: minimum infection rate; PF: positive flocks; C/PF: conversions per positive flock.

From 2000 to 2003, a new approach of WNV risk assessment which analyzed the risk caused by mosquito species for transmitting WNV to human was implemented in New York. This approach associated data related to abundance, infection prevalence, mosquito competence and mosquito biting behaviour. This risk assessment equation can be used as a predictive indicator to assess the relative number of future human WNV infections [180]. 

In 2001, the New York City Department of Health developed the Dynamic Continuous-Area Space-Time (DYCAST) system for WNV risk assessment purposes. The DYCAST model was developed to identify and control high risk areas for WNV in humans at least 13 days before disease onset and was based on dead bird reports to identify high risk areas for WNV transmission. The system used a geographic model based on a localized Knox test to identify the non-random space-time interaction of dead birds, as an index of an intense WNV amplification cycle using biological parameters. This model also related surveillance data with human cases [181]. In 2002, Chicago competent authorities implemented the same system [182]. In 2005, California Department of Public Health introduced DYCAST as a real-time alert system to identify areas at high risk for WNV transmission to humans. Daily risk maps were open access online and used by local authorities to enhance preventive measures (e.g., educational campaigns, integrated mosquito management and enhanced personal protection) in a successful and timely way [183].

In 2002, a new early warning system for WNV was implemented in New York. Mostashari and his colleagues used the SatScan which is a spatial scan statistic model for potential detection of infectious disease outbreaks, applied the data of dead birds reported from New York City and evaluated its effectiveness in providing an early warning of WNV activity. All dead bird reports, mosquito traps and human cases with contact details were geocoded to a point location. Using spatial-temporal cluster analysis of dead bird reporting data, competent authorities can start early larval control activities, prioritize birds for testing and triage scarce mosquito-collection and laboratory resources [184]. 

From 2003 to 2005, a research team of Wyoming (USA) used a spatial analysis tool to estimate potential WNV activity using a spatially explicit degree-day model. This model is based on GIS tool and uses temperature data, which are open access to public and user friendly software. By keeping away the areas without elevated ambient temperature, this model can predict high risk areas for WNV activity [185].

In Colorado, a research team used epidemiological data from 2003 and 2007 for human WNV in order to quantitatively assess: (1) the degree to which spatial scale of data aggregation affected vector borne disease occurrence and (2) the extent of concordance between spatial risk patterns based on disease case counts *versus* disease incidence for regularly used spatial boundary units. Maps based on spatial distribution and correlation of WNV were presented [186]. 

In Suffolk County (New York, NY, USA), a case-control approach was developed since 2005. This approach was based on the association among WNV risk, habitat, landscape, virus activity and socioeconomic parameters and used GIS analytical tools to develop WNV human risk maps [168]. 

In 2005, a research team of Montana State University (USA) developed a human-health risk assessment approach for WNV and associated documented health effects from WNV infection with insecticides used to control adult mosquitoes. Human cases and exposure to adulticides were used as basis of this model. The results pointed out that human health risks from exposure to mosquito adulticides are very low and are not possible to exceed concern levels [187]. 

A research team of Carroll College (Montana) uses another approach of WNV risk assessment model in which spatial and seasonal WNV transmission risk is estimated throughout Montana with GIS models based on the temperature threshold below which virus development will not proceed in *Culex tarsalis*. This model uses maximum, minimum and average daily temperature data; a degree day modeling source that produces WNV development units (WDU) and varying time-temperature scales throughout June, July, August, and September [188]. 

## 4. WNV in Animals

### 4.1. WNV in Birds

#### 4.1.1. WNV Transmission Cycle and Birds

WNV is locally maintained and dispersed in new areas primarily via an enzootic cycle of proliferation, which includes wild and domestic birds as reservoirs and ornithophilic mosquitoes as virus vectors. Apart from birds, the virus is transmitted to dead-end hosts as well [61]. Mosquitoes become infected by feeding on birds that carry virus particles in sufficient concentrations in their blood. Virus introduction and transmission in a non-endemic area, initiates in a wild ecosystem cycle, which involves migratory birds and then in a suburban and urban cycle, involving additionally endemic avian species. Variations in abundance and species composition of birds (species richness) either periodically (e.g., migration period) or irregularly, affect the transmission dynamics of WNV. When species richness increases, a decrease in the prevalence of WNV disease is observed while the disease prevalence increases when species richness decreases [189].

After the dramatic spread of WNV in the USA, with an initial outbreak in New York in 1999 [190] and the upsurge of human cases in 1996-1997 in Romania, with 393 confirmed cases in humans [72], many studies have been conducted to determine the precise role of birds in the epidemiology and transmission dynamics of the virus. A key conclusion that has been drawn from various experimental studies is the great diversity in the profile of viraemia among the different animal species. With an estimated limit of 10^5^ plaque forming units (PFU) per mL of blood, above which the virus can be transmitted to a mosquito during a blood meal, different bird species can develop sufficient viraemia titers for a few days (usually one to four) in order to allow the transmission of the virus to the feeding mosquitoes. These birds belong to the orders of *Passeriformes* (corvids, sparrows, finches, *etc.*), *Charadriiformes* (woodcocks, gulls, *etc.*), *Strigiformes* (owls, eagle owls *etc.*) and *Falconiformes* (various falcon species) [191]. In contrast, species of the orders of *Piciformes* (woodpeckers), *Columbiformes* (doves, pigeons *etc.*) and *Anseriformes* (ducks, geese, *etc.*) develop lower viremia titers, in many cases insufficient to transmit the virus in mosquitoes and they do not contribute in the epizootic cycle.

The direct transmission of the virus between infected and healthy birds kept in the same place, without the presence of vectors, is also of importance [192]. The fecal-oral route was considered as the most probable mode of transmission. The presence of the virus in the feces was confirmed at least in 17 out of the 24 species tested and in the oral secretions of at least 12 out of 14 species [191]. Therefore, transmission through ingestion and/or in mutual grooming of the wings between birds is possible. Transmission of the virus was also achieved by feeding crows with tissues obtained from an infected dead sparrow. These data are particularly important considering that it is difficult to attribute such extensive spatial spread of the virus within a few years, as happened in North America, only to the infection of mosquitoes. Regarding mosquitoes, a minimum period of 10-14 days after infection is required to further transmit the virus to a new host. This time interval is necessary for the virus to replicate in the intestinal cells before invading other tissues and eventually reach the salivary glands, from where it can be transmitted [193]. Therefore, it is very difficult to impute the spread of the virus at 70 km average per month for 6–8 months each year, only to the action of mosquitoes [194].

The involvement of endemic birds and direct transmission of the virus among them constitutes a complementary mechanism of virus spread. Avian species such as corvids are feeding on corpses of animals, including infected dead birds of similar species. They are social, forming large colonies, with a daily dispersal range of up to 20 km from roost, with their area of operations overlapping with the areas of activity of birds from neighboring nests [195]. Also, the young birds remain close to adults and assist their parents in raising their chicks. It is therefore conceivable that birds dying from the disease may be consumed and the virus can be transmitted to birds of neighboring nests. Feeding with corpses even of infected mammals is also a likely mean of viral entrance in a flock. Lastly, the transmission can be achieved orally on chicks. Epidemiological models support these assumptions and the apparent important role of endemic birds in the spread of the virus [153].

WNV outbreaks mainly occur during July-September, in areas that combine the element of water with the presence of cities and a hotbed of resting for migratory birds. Migratory flocks during spring migration in April and May can carry the virus from endemic areas of Africa and introduce it in places of rest and breed in Europe. There, a cycle of virus amplification takes place for 2–3 months between resident birds and mosquitoes, which results in a massive human exposure to the virus during the summer months. The engagement of resident birds seems necessary to justify, as mentioned above, the spread of the virus to neighboring regions. If this did not occur and only migratory species were involved in enzootic cycle of the virus, its spread would have been, territorial, presenting the image of “frog steps” with outbreaks spread apart at least 300–400 km, which is only rarely observed.

All these characteristics are evident in the outbreaks in Greece. The first outbreak occurred in Central Macedonia, an area with significant water bodies, resting area for migratory birds, with not only abundant presence of mosquitoes of the genus *Culex*, but also significant presence of people and great biodiversity of endemic birds. The exposure of the endemic birds of the area was both serologically and molecularly confirmed [46]. However, the genetic similarity of the corresponding virus with isolates in Hungary and Austria in previous years [34,37] supports the assumption that the virus spread southwards.

The autumn migration of birds from Northern Europe to wintering areas in Africa with resting stops in Greece, offers one such possible mechanism, which is supported by the detection of antibodies to WNV in young migratory birds (turtle doves), born in northern Europe, on their arrival in Greek territory. These birds could only have been exposed to the virus in the country of their origin, and this supports the hypothesis on possible entry of this strain during the past, in a similar manner. The subsequent spread of the virus, especially to Southern Europe, is combined by a correlation between areas with human cases and seroprevalence in endemic birds. Seropositive birds have been found only in areas where outbreaks in humans have been recorded. In contrast, in survey it was found that all birds coming from areas with no reported human case were seronegative. In the region of Attica, samples obtained from a period prior to the onset of cases in humans were seronegative, indicating a possible later introduction of the virus in the region, and therefore concerns were expressed that the multiplication cycle of the virus (bird-mosquito-bird) is in its very beginning, expecting a significant increase in human cases, which unfortunately was confirmed for the region [173].

The prevailing opinion on the introduction of the virus in North America involves migratory birds as carriers of the virus during their migratory journey. Indeed, this hypothesis is strengthened by the emergence of a new virus outbreak in Florida, two years after the outbreak in New York, at several hundred kilometers south. The most likely explanation is the transfer of the virus (“frog steps”) by infected migratory birds during the autumn movement to the south along the eastern coast of the USA [195]. 

The short duration of viremia in birds, which is sufficient for the transmission of the virus via mosquitoes, raises questions about the real possibility of birds to carry the virus to new long distant areas. However, it is known that migratory birds tend to travel very long distances of hundreds of kilometers, for several hours (up to 10 hours) during one night only, at speeds that reach up to 100 km/h. Therefore, it is very likely that for an infected bird to reach a resting area, or its destination during the 48 to 64 hours after infection when viremia titer is usually sufficient for the transmission of the virus in mosquitoes fed on the blood of the viremic bird. The arrival of migratory birds just before dawn, when mosquito activity is at peak, further supports this hypothesis. The transmission of the virus in the following days in sedentary birds of the area leads to the onset of a local enzootic cycle of WNV.

The above data demonstrate the multi-factorial nature of the transmission cycle of WNV with a multitude of different climatic, environmental and ecological factors contributing to the progress of the disease spread. At the same time, many avian species seem to be able to play an important role in the epidemiology, a fact that calls for a thorough and continuous investigation.

#### 4.1.2. The Disease in Birds

The introduction of WNV in North America in 1999 was accompanied by high mortality in several species of birds. In the USA, more than 300 bird species have been identified as susceptible to WNV, with high rates of mortality (http://www.cdc.gov/ncidod/dvbid/westnile/birdspecies.htm). The population decline of various species was considered a result of infection from WNV, with the typical example of the decrease of the numbers of American crows by 45% since the entry of the virus in the New World [196]. Extensive studies on postmortem examination of birds and experimental infection with a pathogenic strain NY99, have demonstrated extensive distribution of the virus to the CNS and in peripheral organs, and the highest titers of viraemia in some cases exceeded concentrations of 10^10^ PFU/mL (e.g., to American crows) [191]. 

More specifically, after experimental infection of American crows with the NY99 strain, birds showed signs from the fourth day after inoculation. Many signs such as depression, anorexia, decreased defecation, inability to fly, gait disorders, as well as bleeding from the mouth and the cloaca were reported. Death occurred 24 hours later [191,197]. Similar signs occurred in other species (magpies and other corvids, sparrows, finches, *etc.*) with differences in the day of appearance of signs and outcome of the disease (4–10 days after inoculation). In contrast, other species did not show any signs, and particular species belonging to classes of *Piciformes* (woodpeckers), *Columbiformes* (pigeons, doves *etc.*) and *Anserinformes* (ducks, geese, *etc.*), demonstrating the need for a different approach of the impact of WNV infection per species.

Sensitive species exhibit macroscopic and histopathological lesions such as congestion of the cerebral cortex, the spleen (splenomegaly was observed in some cases), heart and kidneys, dotted hemorrhages in the stomach, liver and skeletal muscle, gallbladder distension and opaque air sacs. Histopathological examination revealed the presence of hepatocyte necrosis with cytoplasmic vacuoles, rupture of the nucleus and strong presence of mononuclear phagocytes in the tissue. The congestion of the spleen is accompanied by lymphocyte necrosis in the lymphoid follicles and increased presence of inflammatory cells, especially macrophages throughout the splenic parenchyma. Skeletal muscle fibers were inflated with loss of striations and fragmentation of sarcomeres, while necrosis of the bone marrow has been also observed [198].

Virus isolation from various organs is possible, both in symptomatic and asymptomatic bird species. The virus has been isolated from the spleen, kidney, brain, heart, lungs and trachea from various species. The relatively easy isolation from feathers and skin samples is particularly interesting and enhances the probability of transmission to mosquitoes, while the isolation from the ovaries indicates a possibility for vertical transmission [191].

In contrast to the New World, the mortality of birds in the Old World seems to be limited [59,199,200], although during the peak incidence in Israel 1997–2000 a large number of storks and geese died due to infection with lineage 1 virus strain [201]. In 2003–2004, two different strains of WNV of lineage 1 and 2 type caused fatal encephalitis in a flock of geese and various falconids, respectively, in Hungary [34,202]. In 2008, a strain belonging to lineage 2, similar to that of Hungary, resulted in death of six falconids in southeastern Austria [37]. From 2001 to 2007, WNV was identified as the cause of death of golden eagles (*Aquila chrysaetos*) and Spanish eagles (*Aquila adalberti*) which showed neurological signs in Spain, while a recent experimental study demonstrated the susceptibility of European partridges to the virus with a viremia titer greater than 10^7^ PFU/mL, which is sufficient to transmit the virus to mosquitoes [203]. In contrast, the experimental infection of a hybrid falcon (3/4 *Falco rusticolus* X 1/4 *Falco cherrug*) revealed the resistance of them, since no clinical signs were observed, and the highest titer of viremia recorded was 10^3.8^ PFU/mL, which is insufficient for the transmission of the virus to feeding mosquitoes [204]. It is therefore evident that there is a great diversity in the observed clinical signs among different bird species, as well as among similar bird species of different regions in the world, which is probably related to the history of exposure to the virus and the different immunological status of the infected birds in each case. 

#### 4.1.3. Surveillance of the WNV Circulation in Greece Using Domestic Birds

Following the onset of WNV epidemics in the USA, a variety of arbovirus surveillance systems were developed. These systems included avian-, mosquito-, and nonhuman mammal-based sampling, to facilitate prediction and prevention of human and domestic animal infections [40]. Similar arbovirus surveillance systems have sporadically been used in Europe, but there is insufficient evidence for their value as early warning indicators for WNV infection [205,206,207]. Moreover, given that in Europe a variety of circulating viral strains of different virulence exists, it is important to follow a surveillance strategy, which not only would allow for early serological detection of the circulation of the virus, but would also allow for isolation and molecular characterization of the circulating strains. Such arbovirus surveillance systems may be particularly useful for better public health risk assessment, and for proper guidance regarding the implementation practices for mosquito control. 

Since the beginning of WNV epidemics in Greece in the summer of 2010 and until 2012, different surveillance systems were developed and constantly evaluated, aiming to monitor WNV circulation mainly in the Region of Central Macedonia, and also in other areas of Greece [208]. These systems include WNV active surveillance, using domestic birds, and particularly, pigeons and chickens. 

A serological study was conducted in domestic pigeons (*Columba livia domestica*), aiming to investigate their suitability as sentinels for WNV surveillance, for the assessment of the geographical spread of WNV after the epidemics of 2010 and 2011 in Greece, and also their effectiveness at alerting upon WNV enzootic circulation prior to the onset of the 2011 and 2012 epidemics in humans. WNV circulation was detected in pigeons 1.5 months prior to the onset of the epidemic in humans. Another study was conducted in free-ranging chickens, younger than five months old, during 2011 in Central Macedonia, with similar results [208]. 

Furthermore, a serological study in captive sentinel chickens was conducted in Central Macedonia, between May–October 2011 and 2012, in order to detect local enzootic transmission of WNV prior to the onset of WNND cases, resulting in detection and molecular characterization of the circulating strain, and early warning. Specifically, from May 2011, blood samples were being collected on a weekly basis from chickens which were placed in cages in the perimeter of Thessaloniki city. The samples were subjected to serological and molecular testing. In parallel to blood samplings, mosquito populations in the study area were monitored using CDC light traps [47]. The study was repeated in the greater region of Central Macedonia during 2012 [209]. The first seropositive captive chicken was detected one month prior to the first WNND case at the same area for 2011. The virus was isolated from the blood of two sentinel chickens and the genomic region of the NS3 protein of both isolates presented highest nucleotide sequence identity (99.9%) to the strain “Nea Santa-Greece-2010”, responsible for the 2010 Greek epidemic. The inferred NS3 amino acid sequences were 100% identical, maintaining the amino acid substitution H_249_P, which might be associated with increased virulence of the strain [47]. For 2012, nine WNND cases were detected in the areas which were monitored, after the detection of the first seroconverted captive chickens. The detected strain presented highest nucleotide sequence identity (99.8%) to the strain “Nea Santa-Greece-2010”, and only three synonymous nucleotide substitutions consisting of transitions were identified, indicating minimum evolution of the virus during 2010–2012 [209].

The development and implementation of the aforementioned systems resulted in informing the public health authorities, in order to take timely measures to protect public health in response to the WNV epidemics of 2011 and 2012. The results regarding WNV enzootic circulation were communicated to the Hellenic Ministry of Rural Development and Food, the Hellenic Ministry of Health and Social Solidarity, as well as to the Hellenic Center for Disease Control and Prevention.

In conclusion, it appears that determination of juvenile seroprevalence in domestic pigeons and free-ranging chickens can comprise reliable and cost-effective systems for early detection of WNV enzootic circulation. With these systems, it became possible to study the spread of the virus in an area after the end of the epidemic. However, it should be noted that only a limited number of blood samplings was feasible to perform. On the other hand, since purchase, maintenance and care of these birds is not required (performed by the bird owners), these systems are very practical and inexpensive for obtaining information on WNV circulation. On the contrary, the application of WNV surveillance systems using captive chickens is more expensive, as bird purchase, accommodation and feeding costs are required. There are several advantages of captive chicken surveillance, compared to the previous system, because it gives the opportunity for repetitive blood sampling, aiming not only to study the geographical spread of WNV using serological methods, but also to the detection, isolation and molecular characterization of the circulating strains. The use of captive chicken with repeated sampling is particularly useful in areas where many WNV strains of varying virulence co-circulate, and collection of this data is imperative in order to accurately determine the risk of human infection and the outbreaks of WNND cases during the epidemic period.

#### 4.1.4. Surveillance of the WNV Circulation in Greece Using Wild Birds

A project for the collection of specimens of wild resident and migratory birds active since 2009 has been running in Laboratory of Microbiology and Parasitology—Department of Veterinary Medicine, University of Thessaly in the context of participation in a research programme of the EU on the surveillance of wildlife diseases (FP7, WildTech). The project was based primarily on the collection of tissue and serum samples of hunter-harvested birds collected during the hunting seasons, but also on passive surveillance using samples obtained from dead birds of unknown etiology. With the onset of the epidemic of 2010, the project was intensified and included surveillance of both archived and the new collected samples for the presence of WNV or WNV specific antibodies. The laboratory investigation involved the molecular detection of the presence of virus by means of RT-PCR, using specific primers that target the genomic region of the NS5 protein and the detection of specific antibodies using competitive ELISA (cELISA), IFAT and confirmed using the serum neutralization test (micro-VNT). By testing of archival samples, the exposure of sedentary wild birds (Eurasian magpies) to WNV was detected at least eight months before the first report of human cases [210]. In collaboration with Hunting Federations, tissue samples and sera of hunted birds were collected from the region of Central Macedonia and the rest of Greece during the hunting seasons 2010–2011 and 2011–2012. Priority was given to search corvid samples, which have been recognized as important indicators of the presence of the virus locally, and samples of migratory birds that have been identified as carriers of WNV and meet the above requirements to transfer the virus over long distances in migration, when they are in a viremia stage. The detection of WNV antibodies in young migratory birds on their arrival in Greece support the hypothesis described in Section 4.1.1. 

### 4.2. WNV in Equids

#### 4.2.1. Introduction

Horses and other equine species are susceptible to WNV infection [211]*.* Unlike birds, horses as well as humans are incidental hosts of WNV [211,212]. Phylogenetic studies performed in WNV isolates obtained from equines have led to their classification into two main lineages [31,36,213,214].

Horses are characterised as dead-end hosts for the virus, since they do not develop titers of viraemia sufficient to infect mosquitoes and propagate the virus [31,36,213,214,215,216]. Horses, as opposed to wild birds and mosquitoes, do not perform an essential role in the spread of the virus. Direct transmission of WNV from horses to humans has been reported very rarely and only in cases handling infectious brain without special precautions during necropsy of dead horses [212,217,218].

In temperate zones of the northern hemisphere, disease risk appears to be greatest during a 3-month period, between August and October, when mosquito activity peaks, whereas in subtropical regions the period of risk is longer and the outbreak pattern may not be predictable and cases may appear year-round [212,216].

Epidemiological studies during epidemics of WNF have identified important risk factors that predispose to infection of horses. Appropriate soil and climate conditions for mosquito breeding (backwater, frequent rainfalls, high temperatures) and the presence of domestic and wild bird species allowing ample access to blood meals, could be responsible for an increase of mosquito populations among areas where horses live. This can lead to the easy spread of WNV, after the virus enters the ecosystem usually via migratory birds [212,219,220].

Since the mid-1990s WNV outbreaks have become a major infectious disease problem in horse populations in Mediterranean countries of Western and Central Europe. In the late summer of 1999, WNV infections were recorded for the first time in the Western Hemisphere when 20 confirmed cases in horses occurred in New York State. WNV spread further to all the USA, Mexico, Canada and the Caribbean during the following three years with thousands of laboratory confirmed equine encephalitis cases [217,219].

#### 4.2.2. Pathology

##### Pathogenesis

The pathogenesis of WNV infection in horses is not completely understood [211,217]. The infection is initiated after the inoculation of the virus into the skin by infected mosquitoes. The virus then replicates in local tissues and in regional lymph nodes and is transported via lymphatic vessels to the blood stream. (Langerhans cells of the skin have been associated with this virus transport to the lymph nodes in WNV infections in mice). This virus replication and viremia may seed infection in extraneural tissues, increasing the virus titer in blood and perhaps preceding the invasion of the CNS [217].

Neuroinvasion pathways for WNV are not well defined, but may involve passive diffusion across the capillary endothelium, virus replication in endothelial cells and budding of virus into the CNS parenchyma, or retro-axonal virus transport of infected neurons of the olfactory epithelium [211,217]. The low virus titer and short duration of viraemia in horses, as opposed to birds, as well as the failure to detect the antigens of WNV in vascular endothelial cells make the first two neuroinvasion pathways unlikely to occur [215,217,221].

The mechanism of neurological damage is uncertain [211,217]. In a WNV infection model in hamsters it was observed that many degenerating neurons underwent apoptosis but this was not associated with inflammatory cells, so it was suggested that cellular damage was caused by WNV infection [217]*.* In contrast, it was suggested that neurological damage in natural WNV infection of horses has an immunopathological component, since inflammatory changes were present in the absence of abundant detectable WNV antigens [217,221]*.*

##### Clinical Signs

Equine infections either from lineage 1 or from lineage 2 WNV strains, are often subclinical, causing seroconversion in the absence of clinical signs [31,36,214,217]*.* In clinical cases there is a variety of signs ranging from transient neurologic deficits to fulminating fatal encephalitis.

Reported clinical signs in horses include fever, paraparesis or tetraparesis and ataxia that may be symmetric or asymmetric, recumbency and evidence of intracranial disease including vestibular or cerebellar ataxia and behavioural changes [31,36,222,223,224]. These signs are not pathognomonic for WNV infection. In many clinical cases affected horses frequently show muscle fasciculation and tremors [31,224]. Analysis of a large outbreak in horses revealed that the most common clinical signs were ataxia, gait disorders, muscle fasciculation, depression, and recumbency [219]*.*

Prognosis for infected horses is variable, with the most important contributing factors appearing to be severity of disease and vaccination status [36,220,225]. It has been found that, as WNV enters for first time in a previously non-exposed equine population, mortality among horses that develop clinical disease may be up to 50%. In contrast, as the disease becomes endemic in the region, the mortality rate among diseased horses is dramatically decreased [211]*.* Sternal and mainly lateral recumbency is a strong predictor of death or euthanasia [213].

Retrospective studies have shown that among horses with clinical disease, the mortality rates vary from low to very high, and in some cases exceed 50% [217,220,223]. These mortality rates include horses euthanized for welfare reasons, because of irreversible course of the disease [217,223]*.* In some cases the mortality rates among horses seem to be influenced by individual characteristics relating to each affected animal, such as nutritional and reproductive status or genetic factors [220,225].

##### Gross Lesions

Typically, in most cases of deceased or euthanized horses there are no gross lesions in the anatomical structures of the nervous system [211,221,224]. However, lesions in some cases are macroscopically evident, with petechiae sparsely distributed throughout the entire rhombencephalon and extending multifocally through the entire spinal cord. These petechiae are especially prominent within the thalamus, the caudal brain stem and the ventral horns of the spinal cord [221,222]*.* Many changes in the internal organs and skeletal muscles can be seen as result of recumbency and other secondary complications with no diagnostic value [213,218].

##### Histopathological Lesions

Histopathological lesions of WNV infection in horses are limited to the CNS [211]. The severity of lesions varies between different cases, which is not always directly proportional to the severity of clinical signs. In addition, vaccinated horses with clinical disease develop very mild and limited histopathological changes [211,225]*.*

Histopathological lesions are detected in the lower brain stem and spinal cord, especially in thoracic and lumbar region. They are more severe and extensive in the grey matter than in white matter. Main histopathological findings are mild to moderate non-purulent encephalomyelitis accompanied by gliosis. Histopathological lesions in extraneural tissues are extremely rare to be found in horses, unlike birds, in which can be found in many internal organs [221,224]*.*

Particularly, histopathological lesions involve the basal nuclei, grey matter of thalamus, midbrain, lower brain stem, and ventral and lateral horns of the spinal cord. In equine WNV cases, lesions can be found in the cortex of the hemispheres and even more rarely in the cerebellar cortex [36,211,221,224,225]*.* The main histopathological findings are multifocal mild to severe perivascular cuffs comprised of lymphocytes and a few macrophages, with mild infiltration of lymphocytes and gliosis in the adjacent neuropil [36,212,221,224,225].

In the most severely affected tissue areas, neuronal degeneration is prominent and characterized by central chromatolysis, cytoplasmic swelling, or cell shrinkage. Small, scattered microglial foci, occasional neuronophagia, and presence of small necrotic areas comprised of macrophages, neutrophils and cellular debris may be seen. Axonal swelling and spheroid formation is frequent [211,221,224]*.*

In addition, in some severe cases mild to severe perivascular hemorrhage with degenerative changes, local necrosis in the vascular wall and the presence of low neutrophil counts may be seen. Occasionally, mild leptomeningitis that extends as mild focal inflammation in the underlying areas of the cerebrum and cerebellum may be found [211,221,224].

##### Distribution of WNV in the Tissues—Immunophenotyping of Lesions

Application of immunohistochemical methods on tissue sections revealed remarkable features for WNV antigen distribution in the nervous system of infected horses and basic qualitative features of inflammatory infiltration [224].

On sites of inflammatory lesions, WNV antigens are mainly localized within the grey matter and have a finely granular appearance within the cytoplasm of a few morphologically normal and degenerate neurons. WNV antigens are also present in a large number of morphologically normal nerve fibres, axonal hillocks, glial cells, and spheroids of the medulla oblongata and spinal cord. No viral antigens have been detected within the peripheral nervous system and extraneural tissues of affected horses, which is in direct contrast to post mortem findings in birds [224].

Perivascular and leptomeningeal inflammatory infiltration is composed almost exclusively of T-cells (labelled for CD3 surface antigen). Occasionally, the perivascular infiltration is composed purely of macrophages (labelled for MAC-387 antigen), and rare macrophages contained intracytoplasmic WNV antigens, whereas, no BLA-36 positive lymphocytes (surface antigen mainly expressed in B-cells) were identified [224].

#### 4.2.3. Diagnosis

##### Ante Mortem Diagnosis

Clinical signs of WNF in horses are not pathognomonic, so the diagnosis of infection is established by laboratory investigation. Detection of WNV in the blood using RΤ-PCR is hampered by the typically short duration and low level of the viraemia in horses. Negative virus detection test results should thus never be regarded as evidence of absence of WNV [36,217,226]. Furthermore, the biochemical and cytological examination of CSF from diseased animals has no diagnostic value, because in many cases abnormal features are not found. *Ante mortem* diagnosis is most commonly achieved by detecting WNV-specific lgM antibodies, using an IgM capture ELISA (MAC-ELISA) [36,215,216,227].

##### Post Mortem Diagnosis

Post mortem diagnosis most commonly involves the use of immunohistochemistry in order to detect WNV antigens in CNS tissues [223,228] as well as PCR testing on CNS tissue samples [213,223,226,228].

#### 4.2.4. Treatment and Prevention

##### Therapeutic Care

Treatment of WNV infection in horses consists primarily of supportive care. No antiviral therapies with documented efficacy against this virus are currently available [222,227]. The supportive care is not always successful [213]*.* However, recent studies have documented clinical improvement in neurologic signs when intravenous immune globulin containing specific antibodies against WNV was administered post infection. The efficacy in animal models appears to be dose dependent; but efficacy studies in naturally occurring equine infections have not been performed [227]. In horses that survive, the period of recovery from the signs until the full clinical cure can last up to two months [36].

##### Prevention—Vaccines

Prevention against WNV infections in horses includes two main actions: to control the mosquito population in the animal environment and to implement vaccination programmes [220,225,227].

Although human vaccines against WNV have not been produced yet but are under production (refer to Section 3.6), five vaccines for horses have been already approved for release in the USA, three of which are available on the market (Table 8). The first was developed by Fort Dodge Animal Health, a subsidiary of Pfizer-Zoetis. The vaccine consists of whole viral particles of lineage 1 inactivated in formalin. The brand name of the vaccine is West Nile-Innovator^®^. It is considered particularly effective because the two doses over 12 months provide adequate immunity in the 94% of animals. Just 5% of horses vaccinated in a safety trial had some side effects [229]. The vaccine is marketed in Europe under the name Equip (Duvaxyn) WNV. Another inactivated vaccine (Vetera WNV vaccine^®^), which was developed by Boehringer Ingelheim Vetmedica, is approved by the USDA and is marketed. A third vaccine is Recombitek Equine WNV Vaccine (Merial) which is a chimeric recombinant vaccine with Canarypox virus [230]. In these vaccine proteins prM and E of NY99 strain of the outbreak of 1999 in New York are expressed. All vaccines develop satisfactory immunity by inducing the production of neutralizing antibodies [231]. In 2005, a vaccine based on the expression of plasmid DNA (West Nile Innovator DNA^®^—Fort Dodge Animal Health/Pfizer-Zoetis) encoding the proteins prM and E, was approved by the USDA but it was recently stopped being marketed. Finally, a chimeric vaccine (PreveNile^®^/Intervet) containing a vaccine strain of Yellow fever virus (strain YF17d) which imported genes encoding proteins prM and E of NY99 strain, was approved for marketing in 2006 by USDA but in 2010 it was withdrawn from the market as cases of acute anaphylaxis, colic and even death in horses were reported [225,227].

The emergence of pathogenic strains of lineage 2 in Europe raised the question whether the existing vaccines, which are mainly developed based on strains of lineage 1 of North America, are effective in protecting the horses. Studies have shown that Recombitek Equine West Nile^®^ of Merial can lead to the development of adequate immunity in horses to protect them from pathogenic lineage 2 strains [232].

New prospective for the development of a new type of vaccine for use in horses was created by the study of Chang *et al*. using plasmid DNA of the virus which leads to the formation of so-called «single-round infectious particles» (SRIPs), which can infect cells of the body but have the possibility of a single replication cycle without further proliferation. This vaccine induced the production of neutralizing antibodies in horses [233].

In the Greek market no commercial vaccines were officially released until recently and as a result they were being imported from other European countries. At this time, Fort Dodge Animal Health/Pfizer-Zoetis holds a license for the vaccine Equip (Duvaxyn) WNV in Greece.

**Table 8:** Vaccines against WNV for horses.

Vaccine	Antigen	Status
West Nile Innovator^®^, Fort Dodge Animal Health/Pfizer-Zoetis	Whole viral particles inactivated in formalin	Approved and marketed
Vetera West Nile vaccine^®^, Boehringer Ingelheim	Inactivated virus	Approved and marketed
RecombiTek^®^, Merial	prM and E proteins in chimeric *Canarypox virus*	Approved and marketed
West Nile Innovator DNA^®^, Fort Dodge Animal Health/Pfizer-Zoetis	Plasmid DNA	Recalled from the company
PreveNile^®^, Intervet	prM and E proteins in chimeric *Yellow Fever virus*	Recalled because of side-effects
Chang *et al.*, 2008 [233]	Plasmid DNA for the formation of SRIPs	Under evaluation

#### 4.2.5. Surveillance of WNF in Horses in Greece

##### WNV Infection in Horses Prior to 2010

Until 2010 there have been no reports of disease caused by WNV in horses in Greece. However, the presence of mild strains of the virus in Greece had been reported from the 1970s. Seropositive horses and other mammals as well as birds have been found in a study conducted by the Laboratory of Microbiology and Infectious Diseases, Faculty of Veterinary Medicine, Aristotle University [234].

Furthermore, epizootiological studies on various infectious diseases including WNV were carried out during the period 2001–2004. The survey was conducted by the Department of Virology of the Institute of Infectious and Parasitic Diseases, Hellenic Ministry of Rural Development and Food (MRDF), in the context of the preparation of Greece for the Olympic Games of 2004. More specifically, blood sera were collected from 7,549 asymptomatic horses living in Greece or transited through the country and were examined by the method of serum neutralization test for the detection of neutralizing antibodies against WNV. The study revealed the presence of neutralizing antibodies against the virus in 35 Regional Units in the country and ~4% of all tested samples.

During the period 2004–2010, a small scale serological survey for infectious diseases in horses including WNV was conducted by the Department of Virology MRDF, as part of the audit is done before the movements of animals. Antibodies to WNV were detected in 2% of the asymptomatic animals tested. 

##### Diagnosis of WNV Infections in Horses with Neurological Signs during the 2010 Epidemic

During the epidemic of 2010, in addition to the diagnosed human cases of infection, 17 horses with neurological signs were diagnosed for the first time in Greece, by the Faculty of Veterinary Medicine, Aristotle University of Thessaloniki (Laboratory of Microbiology and Infectious Diseases and Companion Animal Clinic). Blood samples taken from the above horses were examined for the presence of specific anti-WNV IgM antibodies with MAC-ELISA. All of the suspected animals were positive for WNV-specific IgM antibodies, evidence of recent infection [235]. The diagnosed horses resided in Central Macedonia (Thessaloniki, Kilkis, Serres, Pieria and Chalkidiki).

##### Clinical and Serological Surveillance of WNV in Horses in Greece (2010–2011)

The Hellenic MRDF has implemented a monitoring programme of WNF in horses and wild birds in the country, in order to investigate the epidemic which occurred for the first time in 2010 and continued in 2011. In particular, for equines, this programme included both passive and active surveillance of the disease.

Active surveillance of WNF consisted, initially, of targeted clinical surveillance on horses from specific farms and then, serological surveillance using sentinel seronegative horses in selected areas. It was implemented in Regional Units (former perfectures) of the country where outbreaks were detected in horses or humans, and the Regional Units bordering to them. All equine farms located in a radius of 20 km from the previously confirmed cases of disease in humans or animals were included. At least one visit was carried out by a state veterinarian in these farms in order to perform clinical examination of horses and gather any available information (by farmers, responsible person of the farm or attending veterinarian), which could be an indication that the animals had signs compatible with disease caused by WNV, as well as any information about animal movements within or outside the country in the past and any relevant laboratory results. If the findings of this investigation suggested either current or previous presence of the disease in the farm (clinical signs before up to 3 months), the farm was characterized as suspicious and the veterinarian had to collect blood samples from all horses alive and tissue samples from every dead horse (if there were any) and send them to the laboratory.

Serological surveillance concerned blood sampling from sentinel horses strategically placed at predetermined locations and at specific time intervals of at least 2–3 weeks. The detection of specific antibodies in a previously seronegative sentinel horse (seroconversion) was considered suggestive of recent virus circulation in the area. Similarly, the detection of WNV-specific IgM antibodies in equines was indicative of recent infection. In case of seroconversion or detection of IgM antibodies in one or more sentinel horses those were replaced at the next sampling by an equal number of seronegative sentinel horses living in another farm within the same region. Results indicated that for 2010 WNV-specific antibodies were detected in horses of Central Macedonia (Chalkidiki, Thessaloniki, Kilkis, Serres) and Karditsa. For 2011, circulation of WNV in horses was detected in numerous areas of Greece (Serres, Karditsa, Larissa, Attica, Preveza, Viotia).

Passive surveillance of WNF concerned all equines (horses, donkeys, mules and hinnies), of the Greek territory. It included the mandatory investigation of each equine case with fever and/or neural/kinetic signs compatible with WNV infection regardless of the area it was found. The investigation was comprised by a visit by a state veterinarian, blood sampling (whole blood, blood serum) and laboratory testing of the collected samples (RT-PCR, cELISA, MAC-ELISA) to the Department of Virology, Institute of Infectious and Parasitic Diseases of the Centre Veterinary Institutions of Athens, MRDF. Results from passive surveillance conducted by the MRDF are available for 2011 and WNV infection was confirmed in 10 horses with neurological signs. Specifically, nine of these horses resided in Attica, while one more horse resided in Thessaly (Karditsa).

The results of the programme for the years 2010 and 2011 have been formally reported by MRDF to the World Organization for Animal Health (Office International des Epizooties, OIE, Paris, France). They have been posted on the website of OIE [236] together with a map showing the areas where the outbreaks occurred, denoting virus spread over vast areas of the mainland.

### 4.3. WNV in Reptiles

Viral diseases in reptiles are being caused by a wide range of viruses classified in different genera and families. Thirty to forty years ago, studies on viruses of reptiles have focused on arboviruses because of their zoonotic importance. Therefore, many retrospective and experimental studies on the Eastern and Western equine encephalitis, and Japanese encephalitis were conducted in reptiles. Over the last decade, outbreaks of WNV infection in humans and farmed alligators in the USA, has resulted in scientific research to focus on species (especially in crocodiles and alligators) that may be susceptible to WNV infection and may constitute the reservoir of the virus [237].

Even though many viruses of reptiles have been recognized, the data for those who are primary pathogens are relatively limited. Experimental studies on the pathogenicity of the virus and the factors involved in disease manifestation are also rare; this is a result of many possible contexts, such as the relatively low commercial importance of reptiles, difficulties with the availability of animals and licensing to use adequate number of animals to get statistically significant results, difficulties with housing reptiles in the site of experimentation or the inability to isolate certain viruses in cell cultures. Viruses as potential causes for immediate extinction of endangered reptiles have redefined the focus of attention of scientists on their genetic characterization, and on the diagnostic methods and pathogenetic mechanisms developed in reptile hosts [237]. When WNV crossed the Atlantic in 1999 and expanded from east to west of the USA, research efforts have been made in identifying alternative hosts (apart from birds) who may be reservoirs of the virus, so the attention has refocused on reptiles. Serological surveys revealed the presence of antibodies against WNV in farmed crocodiles in Israel and Mexico as well as in wild alligators in Florida and farmed alligators in Louisiana in the USA [237]. WNV infection was found to be associated with clinical disease and mortality in farmed alligators in Georgia, Louisiana and Florida, USA. In September and October 2002, a severe epidemic of neurological disease occurred in breeding alligators in Florida. Three American alligators (*Alligator mississippiensis*) with clinically patent disease were euthanises and postmortem examination confirmed WNV infection [237]. The most significant histopathological lesions were modetare heterophile to lymphoplasmacytic meningoencephalitis, necrotizing hepatitis, splenitis, pancreatic necrosis, myocardial degeneration with necrosis, mild interstitial pneumonia heterophile necrotic stomatitis and glossitis. Immunohistochemical staining revealed the presence of antigens of WNV, which was most intense in the liver, pancreas, spleen and brain [238]. Virus isolation and detection of the RNA using RT-PCR confirmed the presence of WNV in blood plasma and tissue samples. Among the tissues tested, the highest concentration of virus (maximum titer 10^8.9^ PFU/0.5 cm^3^) was detected in the liver had, while the brain and the spinal cord had the lowest concentrations (10^6.6^ PFU/0.5 cm^3^ maximum titer for each of the two bodies). The titer of virus in the blood plasma ranged from 10^3.6^ to 10^6.5^ PFU/mL, exceeding the threshold required to infect mosquitoes of the species *Culex quinquefasciatus* (10^5^ PFU/mL); alligators are therefore likely to contribute to the transmission of WNV. Antibodies to WNV were also detected in turtles without developing any clinical disease while experimental infections of green iguana (*Iguana iguana*) and a snake (*Thamnophis sirtalis*) caused their death. 

### 4.4. WNV in Other Species

#### 4.4.1. Mammals with Clinical Disease after Natural Infection with WNV

Clinical cases of WNF after natural infection have occasionally been reported in alpaca, sheep, deer, elk, squirrels, wolves, seals, indian rhinos and primates (*Macaca sylvanus*). Dogs and cats seem to be vulnerable to natural infection but rarely develop clinical disease. Clinical disease has been recorded even in marine cetaceans specifically in killer whale [239].

#### 4.4.2. Mammals with only Detectable Antibodies against WNV but without Clinical Disease

In contrast to the above, WNV antibodies have been detected in many species of mammals, which did not present any clinical manifestations of the disease. This category includes cattle, goats, pigs, deer, bats, badgers, bears, foxes, raccoons, opossums, rabbits, lemurs, primates, small rodents and insectivorous mammals.

#### 4.4.3. Mammals Experimentally Infected with WNV

Several experimental infections with WNV have been performed in various mammalian species. The results have shown that mice, hamsters, cats and rhesus monkeys developed mild to severe clinical signs, whereas rabbits, pigs, dogs, guinea pigs and hedgehogs did not show clinical signs, developing only antibodies against the virus [240,241].

#### 4.4.4. Amphibians

Regarding amphibians, it has been recorded that some species of frogs (*Rana ridibunda, Rana catesbeiana*) may become infected with WNV by developing subclinical to severe clinical disease [242].

## 5. WNV in Mosquitoes

### 5.1. Transmission Cycle of WNV and the Role of Mosquitoes

#### 5.1.1. Introduction

The persistence of WNV in nature is achieved through an enzootic transmission cycle that mainly involves ornithophilic mosquito species of the genus *Culex* and birds [243]. These mosquito species are often referred to as amplification vectors. Mosquito species with mixed feeding habits that carry the virus from the amplification cycle to secondary hosts, such as human, horses and other non-avian vertebrates after feeding on infected birds, are characterized as bridging vectors [69]. However, these secondary hosts are incidental and usually serve as dead-end hosts, since the concentration of the virus in their blood (viremia) is not sufficient enough to infect biting mosquitoes. 

The infection of a suitable vertebrate host with WNV begins with its detection by female mosquitoes. After this initial stage, female mosquitoes search the skin of their host with their mouthparts until they locate a capillary to draw blood. Several bioactive substances, such as analgesics, vasodilators, coagulation inhibitors, and immunomodulators that facilitate blood collection and amplify infection by mosquitoes, are contained in the female saliva and deposited to the hosts during this process [244,245,246]. The final destination of WNV within the female mosquito digestive track, once ingested in a blood meal, is the salivary gland. However, before reaching the salivary gland, WNV virions face a number of challenges that involve successful passage through the proboscis, the oesophagus, the anterior midgut, infection of the posterior midgut and finally dissemination into the hemocele. A comprehensive review of all the obstacles that WNV has to overcome within the body cavity of female mosquitoes is given by Diamond and Blair [247] and Kramer and Ebel [248]. 

When receiving blood containing the virus, female mosquitoes may become infected for all their life span and thus potentially transmit WNV to multiple vertebrate hosts during successive blood meals. Several egg development (gonotrophic) cycles may occur during the life span of a female mosquito, with some species requiring more than one blood meal per cycle [248]. This fact may lead to increased transmission of WNV because 1) contact of infected mosquito vectors with susceptible hosts increases, and 2) blood-feeding has been found to prolong lifespan and boost fecundity of female mosquitoes [248].

Although WNV has been detected in more than 60 species of mosquitoes belonging to at least 12 genera: *Aedes*, *Aedemomyia*, *Anopheles*, *Coquilletidia*, *Culex*, *Culiseta*, *Deinocerites*, *Mansonia*, *Mimomyia*, *Orthopodomyia*, *Psorophora* and *Uranotaenia*, only species belonging to the genus *Culex* are considered to be the major amplification vectors of WNV [59,61,69]. That is because the detection of viral RNA in a mosquito by itself does not necessarily render this species a competent vector, which is a vector that becomes infected with and efficiently transmits the pathogen [247]. A mosquito species may be classified as an important vector of WNV when the following criteria are met [69]: (1) efficient infection of the species and transmission of WNV under laboratory conditions, (2) high relative abundance in the wild species and (3) frequent number of virus isolation from field-collected individuals.

#### 5.1.2. Role of *Culex* Species in the Epidemiology of WNV

The answer to the question “Which is the vector of WNV?” is not a simple one. According to Turell *et al.* [249], the answer depends on both the mosquito fauna of a given area and the relative abundance of the species in that area. However, mounting evidence from a series of studies dealing with population density [180], host feeding patterns [250,251,252] and genetics [253] show that *Cx. pipiens* serves as the most important bridge vector in the northeastern, northcentral, and mid-Atlantic USA, but also in eastern Europe [254] and Russia [255]. *Culex* (*Cx.) pipiens* belongs to the complex known as the “*Cx. pipiens* complex” that also includes three other members: *Cx. quinquefasciatus*, *Cx. australicus*, and *Cx. globocoxitus* [256]. *Cx. pipiens* has also two recognized subspecies, *Cx. pipiens pipiens* and *Cx. p. pallens*, while two separate forms with big differences in ecology “*pipiens*” and “*molestus*” had been recognized for *Cx. p. pipiens* [256]. These two distinct forms are morphologically identical but exhibit extensive differences in their ecology. In particular, the molestus form is considered stenogamous (copulation can occur in confined places), autogenous (first oviposition can take place without a blood meal), non-diapausing (remains active during winter) and mainly anthropophilic. On the other hand, the *pipiens* form is eurygamous (copulation takes place only in open spaces), anautogenous (a blood meal is needed for eggs development), diapausing and primarly ornithophilic [257]. While in northern latitudes *molestus* and *pipiens* are physically separated by occupying underground and surface habitats, respectively, populations of both species have been found to coexist at the surface in southern European regions [258,259,260]. This sympatric occurrence appears to promote hybridization between forms, which may reach 19% of the total population in a given region [260]. Hybrids may be of great epidemiological importance since they display an intermediate feeding behaviour between the two forms promoting the transmission of WNV from the avian hosts to humans. Recent evidences regarding the outbreak of WNV infections in Greece in 2010 suggest an increased role of such hybrid populations on shaping the epidemic [258,260].

The criteria that render *Cx. pipiens* mosquitoes the most important in WNV transmission are the following [256]: (1) increased frequency of infected individuals in the wild [261], (2) moderate efficiency in vector competence for WNV [249,262], (3) high relative abundance in urban areas [180,263], (4) feeding habits that include a wide range of vertebrate hosts (avians, humans, horses *etc*.) [250,251], (5) transmission from infected females to their offspring without infection of the germline cells (vertical transmission) [264], and finally (6) ability of infected females to overwinter and therefore serve as a reservoir of the virus for the next season [265]. Other important vector species in Europe are *Cx. modestus* and *Coquillettidia richiardii*, while *Ochlerotatus cantans* and *Anopheles maculipennis* are reported in Europe as occasional vectors [52,266]. Other important vector species in the USA in areas where they are abundant are: *Cx. tarsalis*, *Cx*. *quinquefasciatus*, *Cx. restuans, Cx*. *salinarius,* and *Cx*. *nigripalpus* [61]. 

### 5.2. Mosquito Management for Prevention and Control of WNV

#### 5.2.1. Integrated Mosquito Management Plan

The most efficient way to prevent and control WNV is through an integrated mosquito management (IMM) programme that utilizes integrated pest management principles (IPM). The strategy implemented in such a programme, is the combination of all available control methods (chemical, physical, biological *etc*.) in the most effective, economical, and safe way to keep mosquito vector populations at acceptable levels [267]. Critical components of an IMM programme designed to minimize the risk of WNV transmission and prevent infection of humans and domestic animals are the following: (1) surveillance, (2) larval habitat reduction, (3) chemical control, (4) resistance management, (5) biological control, and (6) evaluation of adult mosquito control methods [154].

##### Surveillance

A consistent surveillance programme that aims at determining species composition, relative abundance and population dynamics in relation to the climatic conditions prevailing in a specific region, is a prerequisite for effective mosquito control. Mapping of seasonal breeding sites is also critical. During the whole course of the surveillance programme, analytical records of species composition is necessary before engaging in any type of control measures but also for the evaluation of the mosquito control programme. The main components of a mosquito surveillance programme are larval and adult surveillance. 

Larval mosquito sampling in aquatic habitats usually serves as a tool for: (1) estimating the relative abundance of a vector species in a given area and (2) assessing the population density of the target species before and after the application of larvicidal insecticides. The collection of larvae mosquito samples is usually conducted by a team of trained inspectors that also search for new breeding sites. The number of samples taken is usually determined by the size of the surface of the larval habitat; however, a sufficient number of live larvae or pupae have to be transported to the laboratory for species identification by a specialist. Species identification relies exclusively on fourth-instar larvae and earlier instars collected have to be reared up to fourth-instar or adult stage in the laboratory [267]. 

Adult mosquitoes’ surveillance targets in verifying the presence of a mosquito vector species and assess its relative abundance in a given area. Such information is critical for monitoring the activity of potential vector species, setting action thresholds for mosquito control activities, and evaluation of control actions [154]. A wide array of sampling techniques including netting, collection with aspirators, direct human bait catches, suction or traps utilizing attractants (e.g., carbon dioxide, CO_2_ traps) are used for adult mosquito sampling. The efficiency of the sampling method used, depends on several factors, such as prevailing climatic conditions (temperature, relative humidity, wind, *etc*.), the daily rhythm of activity of mosquito species, host-seeking and resting behaviour, and the physiological stages of the mosquitoes [267]. A comprehensive review of available sampling techniques for adult mosquitoes is given by Silver [268]. 

##### Reduction of Larval Habitats

Altering or eliminating mosquito larval habitats represents a permanent, effective and economical method of mosquito control in many areas [154]. This strategy includes single sanitation measures such as proper disposal of used tires, cleaning up of illegal dump sites and rain gutters, as well as more complicated water management projects carried out on a regional scale, such as impoundment and open marsh water management. These efforts may significantly reduce mosquito breeding sites and, therefore, the need of insecticide application in a specific area. 

##### Chemical Control

When reduction of larval habitats fails to maintain populations of mosquito vector species below a threshold level, application of insecticides against either the immature or the adult stage of the mosquito life cycle is necessary. Despite the fact that larvicides may only reduce population densities of mosquito vector species for a short period compared to the reduction of larval habitats, larviciding programmes are considered as an essential component of an integrated mosquito control programme due to their high direct efficiency and target-specific properties. Larviciding aims at keeping the risk of arbovirus transmission low by eliminating a significant amount of the target mosquito population before it reaches adulthood and has the ability to disperse [154]. Since mosquito larvae are often concentrated in limited habitats, the application of larvicides is restricted to smaller areas compared to adulticides. Mapping of breeding habitats represents a valuable tool for effective larviciding, since neglected habitats even of limited size may result in large mosquito broods and the need for large-scale application of adulticides. The recommended compounds and formulations for control of mosquito larvae are given by the World Health Organization Pesticide Evaluation Scheme (WHOPES) [269].

Adulticiding is recommended when accurate surveillance data show that the risk of WNV transmission is high and, therefore, a significant proportion of the adult mosquito population has to be eliminated. The scope of adulticiding operations should be determined according to the following factors [154]: (1) General ecology and topography of the region, e.g. presence of important breeding sites and natural barriers such as rivers, (2) relative abundance, distribution, flight range and age structure of the target mosquito population, (3) time period since the target mosquito species has been found infected with WNV, (4) flight range of bird species that serve as amplifying hosts of WNV, (5) demographic profile (e.g., age structure, labour type) of the human population, and (6) persistence of WNV as indicated by surveillance data. In addition, proper drift of the insecticide applied and the daily pattern of activity of the target mosquito species are necessary in order to achieve maximum control benefits. For example, the optimal period for insecticide application against most members of the genus *Culex* is during night, since these species are nocturnal [154]. Recommended insecticides for space spraying against mosquitoes are given by WHO [270], while a comprehensive review of all the applications techniques utilized in mosquito adulticiding operations is given by Becker *et al.* [267].

##### Resistance Management

A strategy to manage resistance of mosquitoes to insecticides that is necessary in IMM programmes should include [154]: (1) collection of reliable baseline data for programme planning and selection of the proper chemical compounds before engaging in any control activity, (2) detection of resistance as early as possible, and (3) monitoring of resistance on a frequent basis during the implementation of the control strategy. In general, the following three approaches have been suggested for resistance management [267,271]: (1) management by moderation. This approach aims at maintaining the susceptibility genes by using low insecticide rates, infrequent applications and non-persistent compounds. (2) Management by saturation. In this context, an insect’s defence mechanisms are saturated by using sufficient doses of insecticides so that no survivors remain. This is useful during the early stages of selection for resistance genes where these are rare and heterozygous. (3) Management by multiple attacks. In this approach insecticides are applied in mixtures or in rotation in order to exert selection pressures below the level that may lead to resistance. 

##### Biological Control

The use of predators, parasites, pathogens, competitors, or toxins from microorganisms to suppress mosquito populations while avoiding toxic effects in non-target organisms could play an important role as part of an IMM programme. Complete knowledge of the biology of the agent used, as well as its interaction with the ecosystem as a whole is necessary for the successful implementation of biological control measures. Numerous organisms have been used as biological control agents, however the larvivorous fish *Gambusia affinis* represents the best-known aquatic predator of mosquitoes. This species has been extensively used in IMM programmes mainly across the USA and worldwide due to its high reproduction rate, small size and the ability to tolerate variations in temperature, organic pollution, and salinity [267]. Biological control agents against mosquitoes also include: (1) numerous insect species such as carnivorous mosquitoes of the genus *Toxorhyncites,* dragonflies (Odonata), water bugs and beetles (Heteroptera, Coleoptera), and caddisfly larvae (Trichoptera), (2) parasitic nematodes *(Romanomermis calicivorax*), (3) pathogens, such as the entomopathogenic fungus *Lagenidium giganteum,* and (4) mosquitocidal bacteria (*Bacillus thuringiensis israelensis*, *Bacillus sphaericus*)*.* Savopoulou—Soultani *et al.* [272] pointed out that the implementation and sustainability of biological control agents against mosquitoes is difficult compared to chemical methods as predators are unlikely to feed exclusively on mosquito larvae and pupae and effective suppression of mosquito populations by exclusive use of biocontrol methods may take days or weeks [272]. Therefore, biological control may become effective only within the context of an integrated approach.

##### Evaluation of Adult Mosquito Control Methods

Evaluation of control efforts is a necessary component of an effective operational programme against adult mosquitoes. According to CDC, minimum requirements of the evaluation process of adulticiding operations are the following [154]: (1) Estimation of the population density of the target mosquito species before and after the application of the insecticides within and outside the control area by using standard trapping methods, (2) determination of infection rates of mosquito vector species before and after spraying within and outside the control area, (3) monitoring of prevailing weather conditions during adulticiding. If feasible, data collection regarding (1) population age structure of the target species, (2) droplet size and flow rate of Ultra Low Volume (ULV) application equipment, and (3) Global Positioning System (GPS) monitoring of the spray track may greatly improve the evaluation of adult mosquito control efforts [154]. 

### 5.3. Surveillance of WNV in Relation to Mosquitoes

#### 5.3.1. Surveillance of Mosquito Species Infected with WNV

The increasing frequency of human cases reported with WNV in Europe and the Mediterranean Basin has launched large-scale entomological surveys as a measure to monitor the virus spread towards taking timely mosquito control measures to protect public health. Isolations of WNV from mosquito species in Europe are given in Table 9, while mosquito species found infected with WNV in the USA can be found in the CDC web site [273]. In Central Europe WNV was isolated in west Slovakia from *Aedes cantans* [52,274], while pools of infected *Cx. pipiens* mosquitoes were found in Romania [72] and Czech Republic [52]. Reiter [194] reports that WNV was found in 11 pools of *Cx. pipiens*, two pools of *Cx. torrentium* and one pool of *Anopheles maculipennis* in the Danube Delta. In the urban area of Volgograd in Russia two WNV positive mosquito pools, one composed of *Cx. pipiens* and the other of *Cx. modestus* were detected in 2003 [255], while the above two *Culex* species along with *Anopheles messae*, *Anopheles hyrcanus*, and *Coquilettidia richiardii* were also found positive in southern Russia [275]. In the Mediterranean region, the virus was isolated from *Cx. modestus* [276,277], in the region of Camargue (France) after human and equine cases were reported in the same area. In Portugal, *Anopheles maculipennis* [276,278] was found to be WNV positive in an early study while a comprehensive country-wide entomological survey showed that *Cx. pipiens* and *Cx. univittatus* collected in 2004 were found to be infected with WNV [279]. WNV genomic RNA was detected in infected *Cx. pipiens* mosquitoes in 2006 in locations in southern Spain where human cases had also been reported [280], whereas *Cx. perexiguus* collected in southern Spain in 2008 were found to be infected with WNV lineage 1. 

**Table 9:** Isolations of WNV from mosquitoes in Europe(modified from Hubálek 1999 [52]).

Species	Countries
*Culex modestus*	France, Russia
*Culex pipiens*	Romania, Czechland, Bulgaria
*Coquillettidia richiardii*	South Russia, Bulgaria
*Aedes cantans*	Slovakia, Ukraine, Bulgaria
*Aedes caspius*	Ukraine
*Aedes excrucians*	Ukraine
*Aedes vexans*	Russia
*Anopheles maculipennis*	Portugal, Ukraine

A mosquito survey in two northern Italian regions in 2007 and 2008 revealed the presence of WNV in two pools of *Cx. pipiens*. The detection of nine human cases diagnosed with WNND in 2008 in Northern Italy launched a detailed mosquito-based survey in the same region in 2009. This study clearly showed that *Cx. pipiens* is the main vector for WNV in Northern Italy [280]. In Greece *Cx. pipiens* mosquitoes trapped in the region of Central Macedonia where encephalitis human cases were reported a few days before the trapping, were found to be infected with WNV lineage 2 in 2010 [85].

#### 5.3.2. Operational Control Programmes against WNV Mosquito Vectors

Control efforts against WNV mosquito vectors are conducted on regional level and usually coordinated by official state agents and specialized scientists. In the USA, the CDC collaborates closely with the Environmental Protection Agency (EPA) along with federal and local services in issues related to mosquito control programmes. For example, the Monroe County in Pennsylvania initiated a successful WNV Surveillance Programme as a continuation of the Vector Control Programme that was firstly introduced in 1973 [281]. The aim of this project was to minimize and eliminate, if feasible, the risk of WNV disease to humans in the area through a comprehensive Integrated Pest Management (IPM) programme for mosquito surveillance and control. Larval vector populations are monitored in early spring along with natural wet areas, tire sites, storm sewers, and wastewater treatment plants. A network of light and gravid traps is placed in all wastewater treatment plants to monitor adult mosquito populations and samples are sent to the laboratory for WNV isolations. These data are plotted in GIS maps so that the exact location of breeding sites, traps, and samples is known. The control of mosquito-vector populations is mainly achieved through extensive larviciding with products including *Bacillus thuringiensis israelensis* (*Bti*), *B. sphaericus*, and methoprene. Larviciding initiates only when larval densities reach a level of six or more vector larvae per 350 mL, while other factors such as vector competence, mosquito fauna, and human proximity to breeding sites are also taken into account. Adulticiding takes place only in special circumstances or when other control measures have failed. In these cases, ULV spraying with pyrethroids is implemented. This comprehensive and cost-effective programme has resulted in zero human cases being reported with WNV in the region until the end of the year 2002 with the first one reported in September 2003 [281]. 

In the case of an epidemic WNV outbreak, aerial application of insecticides against adult mosquitoes is recommended. Based on very high mosquito infection rates, detection of a large number of dead American crows, and increased human infection risk the Sacramento and Yolo Mosquito and Vector Control District (SYMVCD) engaged in aerial application of pyrethroids in an area of 218 km^2^ in north Sacramento and 243.5 km^2^ in south Sacramento in order to reduce WNV transmission and human exposure to the main mosquito vectors (*Cx. pipiens, Cx. tarsalis*) [282]. The evaluation of these control efforts indicated that aerial adulticiding significantly reduced both mosquito abundance and the quantity of infective bites received by humans in the area. Carney *et al.* [283] showed that the odds of infection after the aerial application was approximately six-fold higher in the untreated area compared to the treated one indicating that aerial adulticiding successfully disrupted the WNV transmission cycle. A cost-benefit analysis by Barber *et al.* [284] showed that these emergency spraying measures are cost-effective if only 15 cases of WNND would need to be prevented. A significant decrease in the abundance of both *Cx. tarsalis* and *Cx. pipiens*, and in the minimum infection rates of *Cx. tarsalis* was also observed after the aerial application of pyrethroids on an area over approximately 215 km^2^ in north Sacramento in 2007 [285]. A three-year study (2004–2006) conducted in the Coacholla Valley, CA, USA tested the hypothesis that mosquito control efforts early in the season may interrupt or delay the amplification cycle of WNV and therefore prevent outbreaks of the disease [286]. Specifically in 2004 control measures started to take place one month after the initial detection of the virus in April and included ground ULV applications of Pyrenone 25-5^®^. These measures had little effect on the dispersal of WNV, since seven human cases were reported later in the season. In 2005 the virus rapidly dispersed in the region after its initial detection at the end of May. Ground and aerial applications of insecticides during May and June achieved limited control against the main vector species *Cx. tarsalis*. However, in 2006 timely aerial ULV applications at North Shore immediately after the first detection of WNV in mid-April effectively suppressed *Cx. tarsalis* populations and limited WNV dispersal by interrupting the early amplification cycle of the virus. The authors of this study attributed the success of the aerial applications to the: (1) ability of the aircraft to cover large regions inaccessible by road, such as shoreline vegetation, (2) coverage of large areas well ahead of the dispersal route of the virus, and (3) frequent treatments (26 applications over a period of 40 nights). Local vector control policies may also have a profound effect on the spread of WNV as a study showed in Chicago, IL, USA in 2002. Within a political and ecological framework, these authors investigated how the different local politics in mosquito control measures implemented by four separate districts (Northwest, Des Plaines Valley, North Shore, and South Cook) may have affected the spread of WNV in the region. Northwest and Des Plaines Valley had been extensively monitoring catch basins as well as running fully operational larviciding programmes in these breeding habitats before the outbreak of 2002. In contrast, the districts of North Shore and South Cook did not engage in larviciding measures until the first human WNV case had been reported. The above four districts also followed different strategies regarding adulticiding. Specifically, Northwest engaged in adulticiding efforts on a frequent basis early in June when the first infected mosquitoes were detected, Des Plaines Valley and North Shore took also some adulticiding action, whereas South Cook took adulticiding measures only when regional health authorities ordered them to do so. These differences led to significant local-scale geographic differences in WNV incidence, with Northwest and Des Plaines Valley reporting only 37 and 14 human cases respectively, whereas 153 and 192 human cases had been reported to the districts of North Shore and South Cook, respectively [287]. These authors attributed this event to the aggressive and timely vector control and educational approach followed by Northwest and Des Plaines Valley districts. 

Recent developments in the EU have led to the foundation of the ECDC. However, a common policy on the control of mosquito-vectors does not yet exist among members of the EU member states and most countries deal with this issue in national, regional or both levels [272]. In Greece operational control programmes against vectors of mosquito-borne diseases are usually conducted on a regional scale. A successful five-year mosquito control programme has been implemented since 2000 in the Regional Unit of Kavala, northern Greece [288]. A significant component of this project was the use of GIS to develop detailed vegetation ecological maps as a tool to predict potential breeding sites. This strategy resulted in only 77% of the total area being treated with insecticides using helicopter and ultra-light motorized aircraft. In this project *Bacillus thuringiensis israelensis*, temephos, and diflubenzuron were used as larvicides, whereas malathion was used as adulticide. A pilot project conducted from 2008 through 2009 in the agricultural area of west Thessaloniki, Greece aimed at assessing the efficacy of ULV aerial adulticiding with two new water-based unsynergized formulations of Aqua-K-Othrin (2% deltamethrin) and Pesguard S102 (10% d-phenothrin) against mosquitoes breading in rice fields [289]. Permits for this research project were obtained from the Greek government and the work was observed by the Ministries of Rural Development and Food, and Health and Social Solidarity, as well as by a European Laboratory consultancy. Novel tools, such as a helicopter with GPS navigation, real-time weather recording, and spray dispersal modeling were used to achieve accurate treatment of the experimental blocks. Mosquito species used for trials were the ones mainly found in the rice field areas of Thessaloniki and these are *Ochlerotatus caspius*, *Cx. modestus* and *Anopheles sacharovi.* Overall, the ULV aerial adulticiding of the two water-based pyrethroids resulted in high mosquito mortality, indicating that their application may successfully suppress high-density mosquito populations as the ones observed in rice fields [289].

Before the WNV outbreak observed in Central Macedonia, Greece in 2010 extensive larviciding measures were implemented for more than 10 years throughout this region [290]. These control efforts are usually conducted between June and August every year and are mainly focused on the flooded rice paddies. Other breeding habitats, such as sewage in villages, are also treated. Larvicides used in these projects were diflubenzuron in the rice paddies and sewage systems, and *Bacillus thuringiensis israelensis* in the natural delta [290]. Immediately after the beginning of WNV outbreak in the region, adulticidal control actions were applied. These efforts included two treatments of pyrethroids (deltamethrin, tetramethrin or d-phenothrin) with an interval of four to seven traps by ULV spraying in villages where WNV human cases were reported. The impact of these measures was evaluated by a network of CO_2_ traps set before and after the application of insecticides. Captured mosquitoes were counted, identified to the genus level, and a random sample of *Culex* spp. from each collection was sent to the laboratory for virus detection [290].

### 5.4. Use of GIS for the Prediction, Prevention, and Control of WNV Mosquito Vectors

#### 5.4.1. Introduction

The main applications of GIS on operational control programmes (e.g., larviciding) against mosquito vectors are the following [267]: (1) determination of potential larval habitats by analyzing digital maps, aerial and satellite photos, and thematic maps, (2) correlation between human disease data and larval breeding sites by spatial analysis, (3) inspection of breeding sites and collection of field data by hand-held GPS devices that allow the accurate and timely update of the main database, (4) timely and accurate prediction of appropriate control efforts based on spatial occurrence of events that may trigger larval development (e.g., river overflowing, local weather data), (5) development of operational maps in order to design a cost-effective control programme, (6) prediction of future potential larval habitats resulting from triggering events by storage and plotting of historical data on operational maps, and (7) accurate application and detailed tracking of control efforts using GPS tools.

#### 5.4.2. Spatial Risk Models

Diuk-Wasser *et al.* [291] developed logistic regression models to estimate the abundance of five mosquito species considered the most likely vectors of WNV in Connecticut, USA in areas where data from adult trapping were not available. The models were developed based on actual data obtained from a trapping network in Fairfield County from 2001 to 2003 and then tested with an independent dataset from the neighbouring New Haven County. Remotely sensed data were used for the extraction of environmental predictors of abundance. The results showed that non-forested areas was a significant predictor for *Cx. pipiens*, surface water and distance to estuaries for *Cx. salinarius*, seasonal difference in the normalized difference vegetation index and distance to palustrine habitats for *Culiseta melanura*, and surface water and grasslands/agriculture for *Aedes vexans*, whereas there were no significant predictors were found for *Cx. restuans*. These data were used for the development of continuous surface maps of habitat suitability for each species in both counties. The production of such risk surfaces may direct control efforts in specific habitats during a WNV outbreak and enhance surveillance of vector species in areas where trapping data are not available. 

The exclusive use of either entomological risk of vector exposure or human case data for the spatial assessment of risk exposure to WNV exhibits significant disadvantages. In the first case, important aspects of human behaviour such as the use of mosquito repellents are ignored, while human epidemiologic data are reliable for risk assessment only when they represent an adequate population base [292]. To overcome the weaknesses of the above two approaches, Winters *et al.* [292] developed a comprehensive spatial risk model for a three-county area in the northern Colorado Front Range. Specifically this model incorporated both the entomological risk of exposure to *Cx. tarsalis* and an epidemiological risk map for WNV disease, but also a novel risk classification index based on independently derived measures of entomological and epidemiological risk. This novel analysis revealed a relatively high risk of vector exposure in the densely populated eastern plains portion of the Front Range compared to cooler areas of the west that have a lower population but used heavily for recreation during the summer season. 

Spatial risk assessment analysis may be also implemented to identify regions of high risk before the arrival of WNV in a specific area. In a comprehensive study, Tachiiri *et al.* [162] developed a sophisticated abundance model of *Cx. tarsalis* and *Cx. pipiens* and combined its output with supplementary data by using GIS and geostatistics tools. This information was used to develop a spatial risk assessment of the potential for WNV in British Columbia, where an endemic activity of the virus had not yet been detected. A wealth of data was used in the study, such as climatic and mosquito observation data, a digital elevation model of western North America, breeding bird population data, and wetland and lake data. The model proved to perform better for *Cx. tarsalis* compared to *Cx. pipiens* and, therefore, risk assessment was only performed for *Cx. Tarsalis*. The results of the mosquito abundance model combined with human population data indicated that the region of Greater Vancouver was the area of greatest risk compared to others. This fact means than an IMM plan should be implemented in this region to suppress populations of mosquito vectors and thus reduce the risk of WNV.

## 6. Integrated Surveillance Systems for WNV

### 6.1. Introduction

The occurrence of WNV outbreaks in many European and American countries led to the necessity for creation and implementation of integrated surveillance systems for the timely and accurate forecast of imminent epidemics, but also for the planning of control interventions. The experience of such programmes is presented below through the description of certain examples from countries that have already applied them.

### 6.2. The Example of California

The most populous state in the USA had already applied an integrated surveillance programme for encephalitis transmitted through mosquito-vectors (e.g., Saint Louis encephalitis) since 1969. The California Department of Public Health, in 2000, one year after the first WNV cases in the USA, proceeded to an extension of the programme in order to include the WNV in it [64].

In California, the first virus appearance was in 2002 with a human case. Then, in 2003, the virus activity was also detected in six counties of the state, when three human cases and one equine case were recorded and the virus presence was verified in dead birds, mosquitoes and sentinel birds. The virus activity enhanced in the following year with the reporting of 778 human cases and 28 deaths [64].

The integrated system implemented in California includes monitoring and analysis of the data concerning climatic changes, but also the virus activity, which is estimated based on the mosquito, sentinel bird, wild bird (dead and alive) and equine testing as well as on the reports of human cases. Therefore, the system is organized based on the following essentials:

(1) Human surveillance: a network of 27 laboratories was established in order to enhance the surveillance efforts. In case of a positive result, an epidemiological investigation of the case is initiated by the local agencies, completing the proper form and then carrying it forward to the central health service of the state [178]. The human cases are not a sensitive indicator for the estimation of virus activity, since the greatest proportion of the patients does not have clinical symptoms, and even if they do, these sympotms usually appear two weeks after infection.
(2) Equine surveillance: due to systematic vaccination of equines, their surveillance does not serve anymore as a sensitive indicator for the epizootic activity of the virus [178]. Nevertheless, equines have been maintained in the surveillance system as the confirmed cases indicate increased likelihood of WNV transmission in the human population of the region, where the infected equine is found [178].
(3) Bird surveillance: there are three ways that birds are utilized in the surveillance system: sentinel chicken trapping and regular blood examination for seroconversion testing, blood collection of wild bird for the estimation of seroprevalence and examination of dead birds (reported by the public).

In 2001, the California system made a step forward in separating recent and chronic infections, deciding to report only the recent ones that could highlight the imminent risk of human cases occurrence [178].

So far, it has been shown that the most sensitive and cost-effective approach is that of regular examination of sentinel birds taken place in strategic points of the examined area, while dead bird surveillance, that was introduced in the California system in 2000, has been proved to be one of the most timely indicators of the virus activity [178], as it is shown in the following figures.

**Figure 9:**
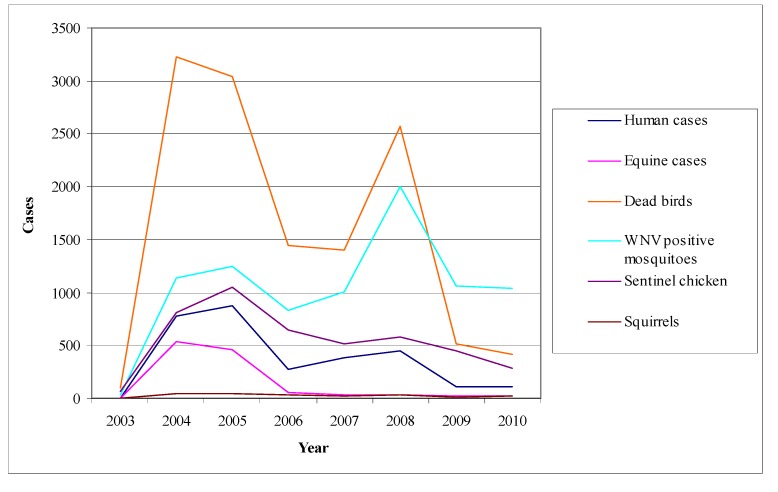
Surveillance system results fluctuation in California, 2003–2010 (data available on: http://www.westnile.ca.gov/ [64]).

(4) Squirrel surveillance: based on indications that squirrels are vulnerable to WNV, the California Public Health Department introduced this parameter in the surveillance system in 2004. This indicator can provide information about a localized virus activity [178].
(5) Mosquito surveillance: although the virus activity detection in mosquitoes is considered to be less sensitive than in sentinel chicken, the mosquito pools examination for the virus presence offers the opportunity of a timely warning system concerning its activity [178]. Even though mosquitoes are not such a sensitive indicator as chicken, their infections may be detected sooner than the seroconversions of chicken. Figure 9 shows the fluctuation of the surveillance system numeric data in California during the period 2003–2010 highlighting two large outbreaks in 2004 and 2008.

**Figure 10:**
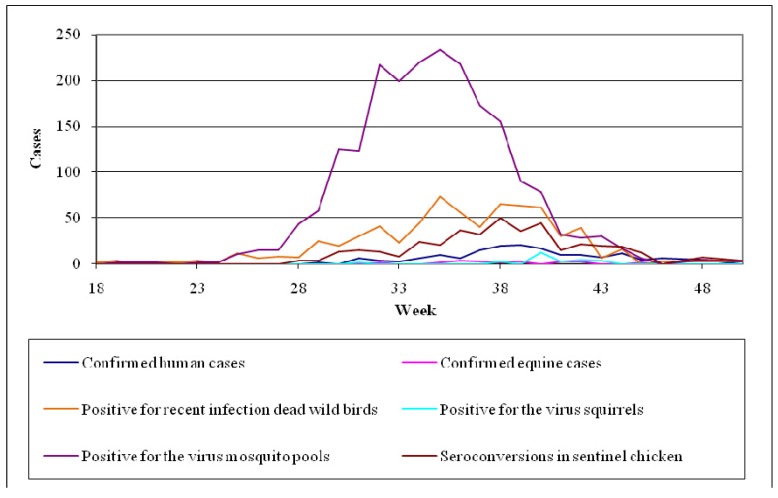
Weekly surveillance data from California, 2011 (data available on: http://www.westnile.ca.gov/ [64]).

Figure 10 shows the weekly surveillance data of 2011. According to Figure 10 the indicators that provide timely warning information seem to be mainly the infection detection in mosquitoes and positive for the recent infection dead birds, as they appear several weeks before the first human case takes place. In chicken, seroconversion takes place just a few weeks before the human cases, while equines and squirrels seem that could not be included in the context of a warning system as they are not a sensitive indicator in the case of California.

### 6.3. The Example of Canada

WNV appeared in Canada in September 2002, when physicians reported a case of polio-like paralysis. During that year, a total of 414 human cases were reported, a number that was 4-fold in the year after [65].

The National surveillance system of Canada is organized including birds, equines, mosquitoes and humans. In particular:

(1) Human surveillance: physicians are alerted to report every suspected and confirmed case; they are supported by a laboratory network in which the samples of any suspected case are sent. In addition, the surveillance system is organized in such a way to ensure the safety of blood units.
(2) Equine surveillance: equine surveillance is under the Canadian Food Inspection Agency in collaboration with Provincial Veterinary Laboratories [65].
(3) Bird surveillance: the Cooperative Wildlife Health Centre of Canada, in collaboration with the Provincial Veterinary Laboratories and the National Microbiology Laboratory, undertake the examination of dead birds from April to the advent of winter. Based on past experience, indicators used are crows, jays and ravens that have been proved vulnerable to the virus and particularly sensitive in determining the extent of its activity [65].
(4) Mosquito surveillance: mosquito surveillance is based on the predictable or existing virus activity, along with the planning of the appropriate interventions. Specifically, in areas with no virus activity, recording and quantification of mosquito species is being conducted, while in regions where virus activity has been ascertained, virological testing of mosquito pools takes place.

**Figure 11:**
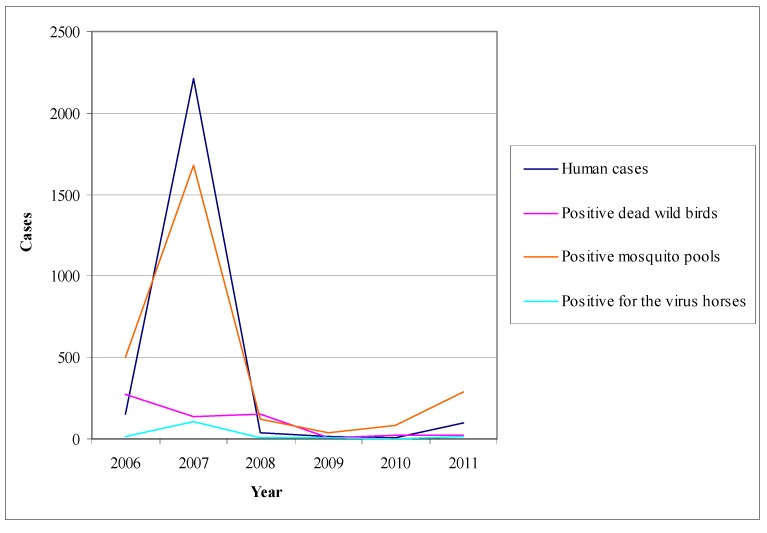
Surveillance system results fluctuation in Canada, 2006–2011 (data available on: http://www.phac-aspc.gc.ca/index-eng.php [65]).

**Figure 12:**
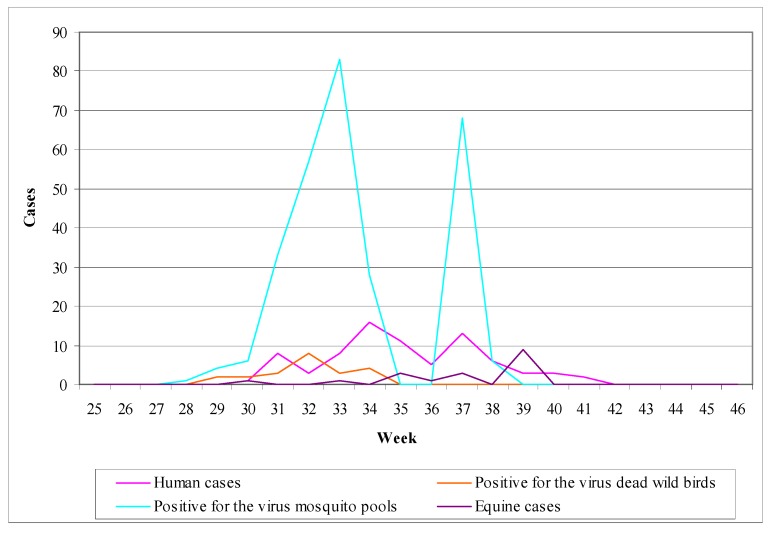
Weekly surveillance data from Canada, 2011.

In Figure 11, where surveillance data of the last six years are depicted, encouraging results of the programme are being highlighted with decreasing with time numbers of cases. In Figure 12, the weekly surveillance data of 2011 are presented. In Canada, the indicator that seems to be the most satisfactory one as timely warning for human cases is mosquitoes.

### 6.4. The Example of Italy (Emilia-Romagna)

The first detection of WNV in Italy took place in horses in Toscana in 1998, followed by an epidemic in horses three years after [68]. In 2001, a national veterinary plan established was aiming at the virus activity surveillance under the coordination of the National Reference Center for Exotic Diseases of Animals [293].

In north Italy, in the Region Emilia-Romagna, a local surveillance programme was launched in 2009. This one was far more intense compared to the national plan as it proceeded to a revision of the surveillance system and organization of entomological surveillance. In particular:

(1) Human surveillance: the main objective of human surveillance was the timely detection of human infections and planning of the appropriate interventions [293].
(2) Animal surveillance: animal surveillance was applied from May to October implemented active and passive surveillance in equine and with active surveillance in non-migratory wild birds [293].
(3) Mosquito surveillance: mosquito surveillance was based on a regular collection and examination of mosquitoes in regions where there were indications of virus activity in humans and animals [293].

**Figure 13:**
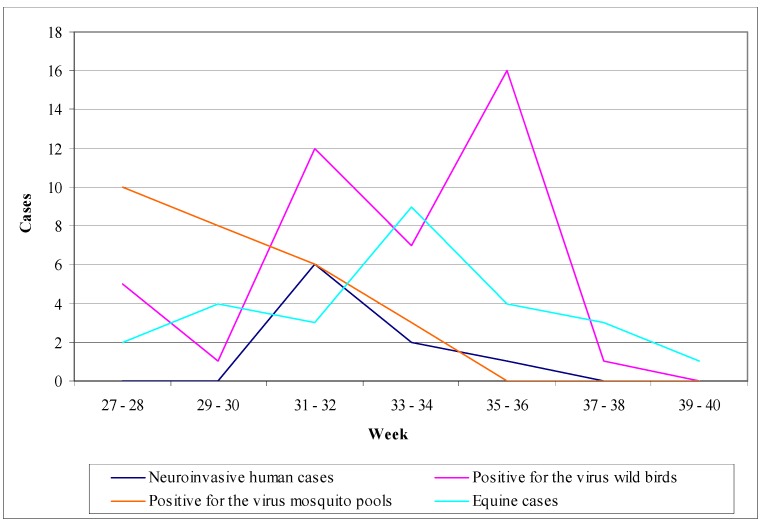
Surveillance data in Emilia- Romagna, 27th–40th week, 2009

In Figure 13, the surveillance data during the first year of the surveillance system implementation in Emilia-Romagna are presented. Even though the Diagram 6.5 highlights the virus detection in equine, birds and mosquitoes before its occurrence in humans, the most sensitive indicator is mosquito surveillance, but surveillance of wild birds is the most timely surveillance approach, and it is also related to fluctuations of human cases.

### 6.5. The example of Romania

The first extensive outbreak of WNV in Europe took place in Bucharest in 1996 causing almost 400 cases and 17 deaths. After this epidemic, Romania proceeded to the implementation of a mild surveillance programme [5].

(1) Human surveillance: during the period 1997–2000, the implementation of enhanced passive surveillance resulted in recording 39 cases including 5 deaths [5].
(2) Chicken surveillance: in 1997, sentinel chicken were used in Bucharest; every week a blood examination was performed in chickens giving an average seroconversion of 24%. During the period 1997–2000, domestic birds were used in Bucharest and in three additional regions in order to estimate virus seroprevalence in birds, giving a result of 8% [5].
(3) Mosquito surveillance: mosquito collection and examination was implemented only in a small extent (16,000 mosquitoes in 1997 and 7,000 in 1999), which resulted negative detection of the virus [5].

Unfortunately, this system was not further intensified and therefore there is no regular detection and recording of positive birds and mosquitoes; therefore, a new outbreak is constantly an existing threat for the country, given the existence of appropriate vectors and vulnerable hosts [5].

Based on the above examples of countries that had or have integrated surveillance systems for WNV, it seems that the implementation of such programmes gives the opportunity of sufficient virus activity detection, sufficient control of the components involved in its activity, timely prediction of its spreading and management of its outbreaks.

In conclusion and mainly based on published data and results coming from the most organized and the most perennial applied surveillance systems (California, Canada), dead wild birds and mosquitoes are presumably the most appropriate indicators for the development of risk assessment models and timely prediction of the occurrence of human cases. In Europe, where numerous strains of varying virulence co-circulate, surveillance of captive sentinel chickens seems to be an efficient approach for human health risk assessment [294], while in Greece, indications from our data have shown that serological surveillance in domestic birds, as well in wild birds, along with mosquito surveillance, has proved to be an efficient approach regarding early detection of WNV circulation prior to the onset of human cases [208]. 

## 7. Conclusions

WNV remains a special pathogen that has gathered much attention due to its spreading pattern around the globe. It has been established in many countries worldwide causing epidemics in humans, birds and equines every year. Several countries of the Mediterranean Basin were the first to be affected after the first virus isolation in 1937, but the most impressively rapid spreading of the virus was observed in the USA starting from New York State in 1999. Studies are continuously conducted to improve the diagnostic tools, discover a WNV specific treatment and invent an efficient human vaccine. So far, only supportive treatment is available, while a number of vaccine candidates are currently being tested with promising results. Despite the scientists’ efforts and the years of experience, WNV remains eventually a new pathogen that has many secrets that need to be revealed.

It has been proved that the persistence of WNV in nature is achieved through an enzootic transmission cycle that mainly involves ornithophilic *Culex* mosquitoes and birds. *Cx. pipiens* is considered to be the most important in WNV transmission due to: (1) increased frequency of infected individuals in the wild, (2) moderate efficiency in vector competence for WNV, (3) high relative abundance in urban areas, (4) feeding habits that include vertebrate hosts, (5) transmission from infected females to their offspring without infection of the germline cells (vertical transmission), and finally (6) ability of infected females to overwinter and, therefore, serve as a reservoir of the virus for the next season. The combination of all available tools (chemical, physical, biological *etc*.) should be implemented in the most effective, economical, and safe way to keep mosquito vector populations at low levels. Critical components of a program that minimizes the risk of WNV transmission are the surveillance, the larval habitat reduction, the chemical and biological control, the resistance management and the evaluation of adult mosquito control methods. Examples of operational control programmes against WNV mosquito vectors that utilize recent advances in GIS, mapping, GPS, as well as novel techniques of spatial and space-time modeling are provided. Detection of antibodies in migratory birds demonstrate the importance of wild bird surveillance for WNV, while wildlife surveillance can be a powerful tool as a part of an effective early-warning system; surveillance in wildlife should be combined with surveillance systems on sentinel domestic birds and horses which have also been proved to be extremely effective. 

More than a decade had passed from the first major outbreak in Europe when Greece was hit by the virus; the first year of the outbreaks was characterized by the highest number of human cases and deaths in contrast to the following years data. Greece implemented a systematic surveillance programme for the responsible vector and the vertebrate hosts that are involved in the transmission cycle; all data derived from this programme were used in risk assessment tool creation in the framework of managing the responsible vector and controlling the spreading in human population. Through the Mandatory Reporting system, the enhanced awareness of health professionals and the active laboratory surveillance for human cases, the HCDCP has managed to ensure an efficient case recording. Greece runs the fourth consecutive year of WNV outbreaks in its territory. Although, the total number of reported cases seems to gradually decline, it is widely accepted that WNV is here to stay and thus annual outbreaks will continue to exist during the following years, while the actual number of cases can never be precisely estimated. Continuous human cases monitoring along with vector control and animal surveillance are required in order to prevent and/or reduce the impact of the disease, recognizing high risk and affected areas early enough in order to implement the appropriate public health protective measures. MALWEST project has achieved an efficient surveillance level in coordination with health authorities, but there is no doubt that without the continuance of such interventions in the future, WNV may follow an unpredictable spreading scenario possibly increasing the numbers of cases and deaths. 
